# *N*-Acetyl-Cysteinylated Streptophenazines from *Streptomyces*

**DOI:** 10.1021/acs.jnatprod.1c01123

**Published:** 2022-04-15

**Authors:** Kristiina Vind, Sonia Maffioli, Blanca Fernandez Ciruelos, Valentin Waschulin, Cristina Brunati, Matteo Simone, Margherita Sosio, Stefano Donadio

**Affiliations:** † NAICONS Srl, 20139 Milan, Italy; ‡ Host-Microbe Interactomics Group, Wageningen University, 6708 WD Wageningen, The Netherlands; § School of Life Sciences, University of Warwick, Coventry CV4 7AL, United Kingdom

## Abstract

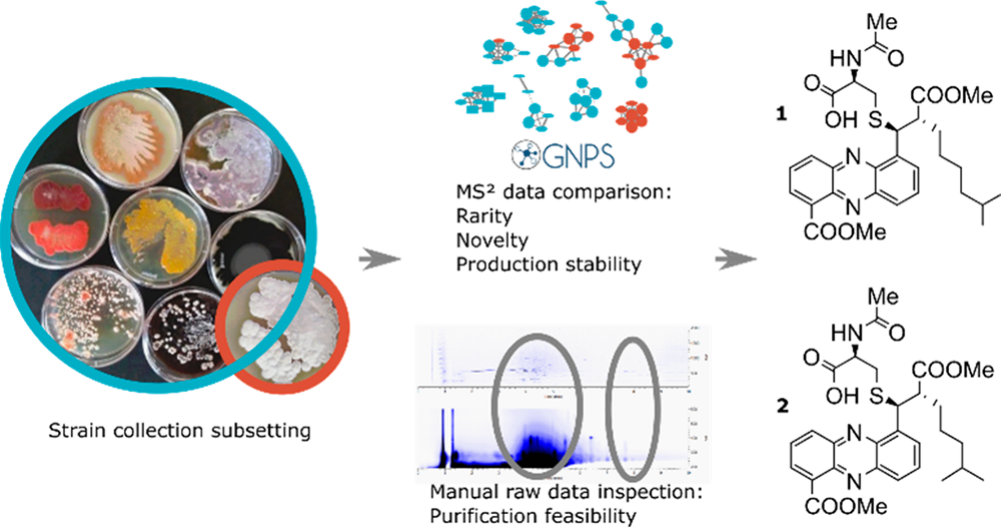

Here, we describe two *N*-acetyl-cysteinylated streptophenazines (**1** and **2**) produced by the soil-derived *Streptomyces* sp. ID63040 and identified through a metabolomic approach. These metabolites attracted our interest due to their low occurrence frequency in a large library of fermentation broth extracts and their consistent presence in biological replicates of the producer strain. The compounds were found to possess broad-spectrum antibacterial activity while exhibiting low cytotoxicity. The biosynthetic gene cluster from *Streptomyces* sp. ID63040 was found to be highly similar to the streptophenazine reference cluster in the MIBiG database, which originates from the marine *Streptomyces* sp. CNB-091. Compounds **1** and **2** were the main streptophenazine products from *Streptomyces* sp. ID63040 at all cultivation times but were not detected in *Streptomyces* sp. CNB-091. The lack of obvious candidates for cysteinylation in the *Streptomyces* sp. ID63040 biosynthetic gene cluster suggests that the *N*-acetyl-cysteine moiety derives from cellular functions, most likely from mycothiol. Overall, our data represent an interesting example of how to leverage metabolomics for the discovery of new natural products and point out the often-neglected contribution of house-keeping cellular functions to natural product diversification.

Resistance has been observed against all established classes of clinically relevant antibiotics,^1^ rendering once easy-to-cure diseases difficult to treat. Hence, there is an urgent need for novel chemical scaffolds with antibacterial activity. With synthetic approaches performing below expectations, natural products are still the prevalent source of antibiotics in human use.^2^ Bioactivity-based screenings introduce bias into natural products discovery, highlighting mostly the metabolites that are both frequently encountered and present in concentrations above the bioactivity test threshold.^3−5^ To expand our knowledge of the vast chemical space covered by natural products, it is necessary to find alternative strategies to identify novel compounds to meet our medical needs.

One of the main challenges in natural products discovery lies in avoiding rediscovery of known scaffolds. The widespread use of mass spectrometry and rapid development of spectrum processing and analysis tools^6−9^ have facilitated recognizing previously described compounds in a process known as dereplication. As databases are biased toward bioactive metabolites and only a fraction of the occurring metabolites have been annotated, only a minority of signals in the metabolic fingerprint of any microbe can be dereplicated automatically. This implies that the potential novelty of an unknown metabolite cannot be deduced from the absence of annotation.

Relying on the assumption that novel chemistry is detected relatively rarely when exploring well-established microbial taxa,^3^ it should nevertheless be possible to pinpoint medically valuable metabolites by exploring a sufficiently large data set of metabolites. In this respect, NAICONS’ metabolic fingerprint library (NMFL), derived from about 14 000 extracts obtained from about 4000 actinomycete strains,^10^ offers a great opportunity for “rarity-based” prioritization of metabolites to discover novel antimicrobials. Recently, a metabolomics-guided approach has enabled the discovery of the unusual biaryl-linked tripeptides produced by some *Planomonospora* strains and permitted the finding that the associated biosynthetic gene clusters (BGCs) are widespread among actinobacteria, although the corresponding metabolites have so far escaped detection.^11^

We describe the discovery of reliably produced antimicrobial streptophenazines featuring an *N*-acetylated cysteine attached to the alkyl chain C-1′ via a thioether bridge through an untargeted metabolomics^12^ approach. We trace the abundance dynamics of these molecules and compare the BGC from the producer strain *Streptomyces* sp. ID63040 to the streptophenazine reference cluster.^13^

## Results and Discussion

### Metabolite Prioritization

**Figure 1:**
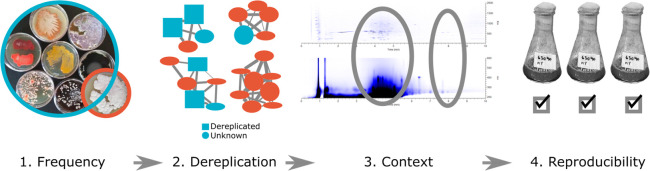
Metabolite prioritization workflow. First, we selected signals absent in the NMFL data set (Frequency); next, we excluded signals associated with annotated molecular families in the GNPS database (Dereplication); then, we manually examined raw data to establish chromatogram crowdedness (Context); and finally, we verified the presence of signals across biological replicates (Reproducibility).

Current results stem from a study aimed at identifying small-molecule elicitors that would trigger production of secondary metabolites. Using 21 randomly chosen *Streptomyces* strains from the NAICONS collection of 45 000 actinomycete strains,^10^ we cultivated them in medium-scale liquid cultures in a single medium but in separate experiments and analyzed the metabolite fingerprints of the corresponding extracts by LC-MS/MS (K.V., unpublished results). During the course of this study, we realized that the available data set could be “mined” for metabolites that fulfilled the following properties: (i) they were not detected in any of the 14 000 metabolic fingerprints contained in the NMFL; (ii) they did not cluster together with any of the annotated metabolites in the GNPS platform;^6^ (iii) they coeluted with few other metabolites by reversed-phase HPLC; and (iv) they were observed in extracts obtained from biological replicates of the same strain performed at different times (Figure 1).

The metabolites occurring in the data set related to 21 strains and absent in the NMFL were considered as rare and potentially novel. The molecular network of 11 362 data files related to roughly 4000 strains and 42 data files related to the set of 21 strains was calculated on the GNPS server (K.V., unpublished results) The resulting table was analyzed as follows. First, we omitted all single nodes, as the presence of structurally related congeners strengthens metabolite identification. Then, we focused on nodes encountered only in the data set of 21 strains. Next, we eliminated signals clustering into molecular families with characterized compounds. This logical flow, which was designed for identification of rarely occurring metabolites that were structurally unrelated to known molecules, led to the identification of 18 molecular families worthy of further investigation.

In the absence of other prioritization criteria, context assessment is a key step for increasing the odds for successful structural characterization. The metabolic fingerprint of a given strain is usually a complex matrix in which not all metabolites can be easily purified due to co-occurrence with structurally unrelated compounds with similar charge, polarity, and/or size. Obtaining enough compound with reasonable purity can be a rate-limiting step in natural product discovery that may require scaling up production and establishing tailor-made purification protocols.^14^ To validate our approach, we gave higher priority to compounds that appeared easy to purify on the basis of the crowdedness of the relevant portion of the reversed-phase chromatogram. Furthermore, in the absence of coeluting compounds, it is possible to unambiguously assign a UV–vis absorption spectrum to the metabolite of interest, which facilitates compound detection during purification. Manual inspection of raw data resulted in prioritization of just two molecular families from two strains.

Finally, to be able to carry out compound characterization using the natural host, it is critical to evaluate the production stability before deciding on which compound to pursue. The metabolites that were produced across experiments (biological replicates with different preparation of the same cultivation medium) were believed to be the result of a robust production process. Both molecular families prioritized in the previous step were present across experiments, representing good starting points for natural products discovery.

#### Isolation and Structure Elucidation of *N*-Acetyl-Cysteinylated Streptophenazines

The metabolites characterized in this paper were obtained from *Streptomyces* strain ID63040. Using LC-MS, we detected two main congeners in the lipophilic part of the chromatogram: *m*/*z* 584.2430 [M + H]^+^ (**1**) and 570.2277 [M + H]^+^ (**2**). Upon fragmentation, we observed neutral losses of 131.0036 Da (for **1**) and 131.0042 Da (for **2**), corresponding to C_4_H_5_NO_2_S (calculated 131.0041 Da), thus establishing the presence of sulfur (Supporting Information Table S1). The calculated molecular formulas for **1** and **2**, corresponding to the observed *m*/*z* 584.2430 (calculated 584.2430) and observed *m*/*z* 570.2277 (calculated 570.2274) [M + H]^+^, were hence C_30_H_35_N_3_O_7_S and C_29_H_37_N_3_O_7_S, respectively.

According to the available records, strain ID63040 was isolated from a soil sample collected near Ziniare, Burkina Faso, on June 6, 1992. Based on its 16S rRNA gene sequence the closest relative is *Streptomyces cellostaticus* NBRC 12849, with a sequence identity of 99.44%. Analysis by autoMLST confirmed the taxonomic assignment (data not shown).

Strain ID63040 was grown in 2.5 L of liquid production medium for 3 days. The culture broth was filtered through Whatman paper followed by EtOH extraction of the mycelial cake and MPLC fractionation, yielding 3.8 mg of **1** as a yellowish-brown, amorphous solid, with UV–vis maxima at 252 and 366 nm (Supporting Information Figure S1).

NMR analysis of **1** in acetone-*d*_6_ established the presence of an *N*-acetylated cysteine residue (Table 1). Moreover, the characteristic UV spectrum and the presence of numerous aromatic carbons with chemical shifts around 141 and 143 ppm suggested that **1** contains a phenazine core. Three hydrogens were detected in each of the aromatic rings. One of the remaining carbons was clearly connected to a carboxylic ester. Additionally, we detected three discrete spin systems: a branched aliphatic chain, one free carboxylic acid, and an additional carboxylic ester (Table 1). The presence of esters was also supported by the indicative carboxyl absorption bands at 1690 cm^–1^ in the IR spectrum (Figure S28).

**Table 1:** Assignments for Compounds **1** and **2** (^1^H 300 MHz, ^13^C 75 MHz)

	**1**				**2**^a^	
	acetone-*d*_6_ at 300 K		DMSO-*d*_6_ at 350 K		acetone-*d*_6_ at 300 K	
position	δ_C_, type	δ_H_, mult. (*J* in Hz)	δ_C_, type	δ_H_, mult. (*J* in Hz)	δ_C_, type	δ_H_, mult. (*J* in Hz)
1	132, C		132, C		132, C	
2	131.3, CH	8.24, dd (6.9, 1.4)	131.8, CH	8.22, d (6.8)	131.3, CH	8.24, dd (6.8, 1.3)
3	129.7, CH	8.03, m	130.3, CH	8.03, m	129.7, CH	8.03, m
4	133.1, CH	8.57, d (8.9)	133.2, CH	8.42, d (8.6)	133.1, CH	8.57, d (8.7)
4a	142, C		141.5, C		142, C	
5a	141, C		140, C		141, C	
6	132, C		132, C		132, C	
7	129.8, CH	8.07, m	130.3, CH	8.07, m	129.8, CH	8.07, m
8	131.2, CH	8.03, m	131.7, CH	7.99, m	131.2, CH	8.03, m
9	129, CH	8.19, dd (8.5, 1.6)	129.2, CH	8.14, dd (8.5, 1.6)	129, CH	8.19, dd (8.5, 1.5)
9a	143, C		143.3, C		143, C	
10a	141, C		141.5, C		141, C	
1′	not detected	5.64, s	45.9, CH	5.42, bd (10)	not detected	5.64, s
2′	51, CH	3.5, m	51.1, CH	3.46, m	51, CH	3.5, m
3′a	31.4, CH_2_	1.5, m	31.3, CH_2_	1.45, m	31.4, CH_2_	1.5, m
3′b	31.4, CH_2_	1.2, m	31.3, CH_2_	1.2, m	31.4, CH_2_	1.2, m
4′	29.2, CH_2_	1.34, m	24.3, CH_2_	1.12, m	26.9, CH_2_	1.18, m
5′	26.9, CH_2_	1.18, m	26.9, CH_2_	1.11, m	38.2, CH_2_	0.9, m
6′	38.2, CH_2_	0.9, m	38.3, CH_2_	0.86, m	27.3, CH	1.26, m
7′	27.3, CH	1.26, m	27.5, CH_2_	1.26, m	21.8, CH_3_	0.67, d (6.6)
8′	21.8, CH_3_	0.67, d (6.7)	22.7, CH_3_	0.65, d (6.6)	21.8, CH_3_	0.67, d (6.6)
9′	21.8, CH_3_	0.67, d (6.7)	22.7, CH_3_	0.65, d (6.6)		
1-COO*Me*	51.8, CH_3_	4.04, s	52.7, CH_3_	4.02, s	51.8, CH_3_	4.04, s
1-*C*OOMe	166.8, C		167.1, C		166.8, C	
2′-COO*Me*	51.2, CH_3_	3.81, bs	51.7, CH_3_	3.7, s	51.2, CH_3_	3.81, bs
2′-*C*OOMe	175, C		174.5, C		175, C	
1″	171.1, C		172.1, C		171.1, C	
2″	52.2, CH	4.8, m	52.3, CH	4.37, m	52.2, CH	4.8, m
3″	33.5, CH_2_	2.96, dd (13.9, 4,5)	34.2, CH_2_	2.82, dd (13.1, 6.1)	33.5, CH_2_	2.96, dd (13.9, 4,7)
		2.84, dd (13.9, 4,5)		2.7, dd (13.1, 8.1)		2.84, dd (13.9, 4,7)
4″(NH)		7.4, bm		7.73, bd (8.04)		7.4
5″	169.2, C		169.3, C		169.2, C	
6″	22, CH_3_	2.05	22.7, CH_3_	1.8, s	22, CH_3_	2.05

a ^13^C NMR shifts were obtained indirectly from HSQC and HMBC experiments.

The number of methyl esters was confirmed by basic hydrolysis with NaOH, revealing one readily hydrolyzed methyl ester (5 min at room temperature) and a second that required overnight incubation (Figure S2). NMR analysis of the hydrolysis mixture showed rapid disappearance of the ^1^H signal at 4.04 ppm associated with ^13^C at 51.8 ppm and possessing an HMBC correlation with the aromatic H-2, indicating that the readily hydrolyzed methyl ester was in the phenazine portion. A similar behavior has been reported that allowed transformation of streptophenazine A into C.^15^ The existence of a free carboxyl group was confirmed by both amidation with ethylenediamine and esterification with MeOH (Figure S3). MS^2^ fragmentation of these derivatives confirmed that modifications were introduced into the *N*-acetyl-cysteine moiety, indicating that the free carboxylic group is located on this moiety (Figure S3).

However, the NMR experiment described above was not sufficient to fully resolve the structure of **1**, as no correlations among the different spin systems were detected in the 2D TOCSY and HMBC experiments. Moreover, out of the 30 carbons from the calculated molecular formula of **1**, one was missing in the NMR spectra and another had a very low intensity. It has been reported that rigid environments can result in missing NMR hydrogen and carbon signals, which can be observed by acquiring the spectrum at higher temperature.^16^ If sulfur was directly linked to one of the missing carbons, it might hamper molecular mobility and lead to missing NMR signals. Thus, we reanalyzed **1** dissolved in DMSO-*d*_6_ with spectra acquired at stepwise increased temperatures up to 77 °C/350 K. Experiments at 350 K allowed observation of the missing proton H-1′ at 5.62 ppm and of the corresponding carbon at 45.9 ppm. These values agree with a thioether substituent at C-1′, the presence of which was confirmed by the NOESY correlation between H-2″ and H1′. Finally, the now visible COSY correlations among protons H-1′, -2′, and -3′, together with NOESY correlations between the aromatic proton H-7 and H-1′ and -2′, established that the alkyl chain was in the same position as in known members of the streptophenazine family (Figures S4–S23). Overall, **1** turned out to be identical to streptophenazine F, except for the OH at C-1′ being replaced by an *N*-acetyl-cysteine moiety linked through a thioether bond.

The absolute configuration of the C-1′ stereocenter was established by measuring the electronic circular dichroism (ECD) of compound **1**. As C-1′ is directly adjacent to the strong phenazine chromophore, the C-1′ configuration is expected to have a major effect on the ECD curve. The presence of a negative Cotton effect at 260 nm indicated an *S* configuration of the C-1′ stereocenter (Figure S24), as in the hydroxylated streptophenazines. The relative configuration of C-1′ and C-2′ was deduced from the ^3^J NMR coupling constant between H-1′ and H-2′, as described for similar compounds.^17^ For compound **1**, the H-1′ signal was missing at room temperature (300 K), but it became a broad doublet with a H-1′–H-2′ coupling constant of 10 Hz when the spectrum was recorded at 350 K. The corresponding coupling constant values described in the literature range from 7.5 to 7.8 Hz for erythro (*S*,*R*/*R*,*S*) to 6.5 Hz for threo (*S*,*S*/*R*,*R*) configurations.^17^ Assuming the presence of sulfur does not affect the coupling constant, the observed value is closer to those for erythro configurations, with the substituents at C-1′ and C-2′ on opposite sides. Thus, the configuration of **1** was established to be C-1′*S*, C-2′ *S* as in other streptophenazines. As discussed below, the cysteine moiety can be assumed to be in the l configuration.

The structure of **2** was deduced from its MS^2^ fragmentation and NMR fingerprint from a sample purified from a 5 L culture as described in the Experimental Section. MS fragmentation data suggested a structure like that of **1** with an aliphatic chain shorter by a methylene unit (14 Da), thus representing a derivative of streptophenazine A in which an *N*-acetyl-cysteine linked through a thioether bond replaces the OH at C-1′. The NMR fingerprint of **1** is superimposable to that of **2** (Table 1), except for the expected missing signals in the aliphatic chain, but featuring the same branched aliphatic chain.

**Figure 2:**
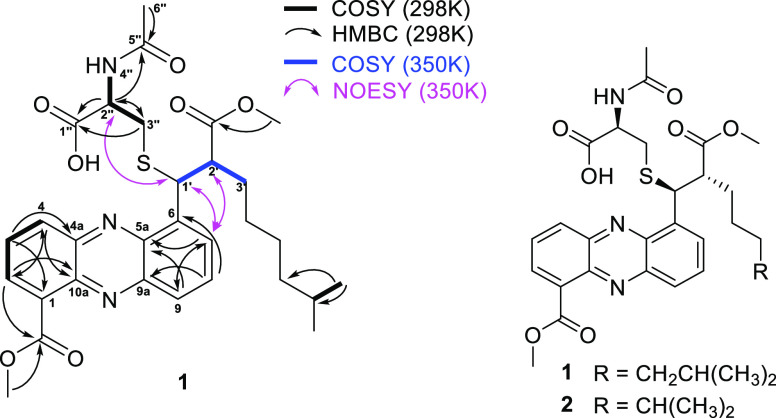
Left: Selected NMR correlations for compound **1**. Right: Structures of compounds **1** and **2**.

In conclusion, **1** and **2** were established to be streptophenazines with an *N*-acetylated cysteine attached to C-1′ of the alkyl chain via a thioether bridge as shown in Figure 2. It should be noted that the nomenclature of streptophenazines suffers from some double booking, as identical names have been assigned multiple times to different metabolites^13,17−21^ (Table S2). For this reason, we prefer to name the compounds reported herein as 1′-(*N*-acetycysteinyl)-1′-deoxystreptophenazines A and F.

### Bioactivity and Cytotoxicity Testing

**Figure 3:**
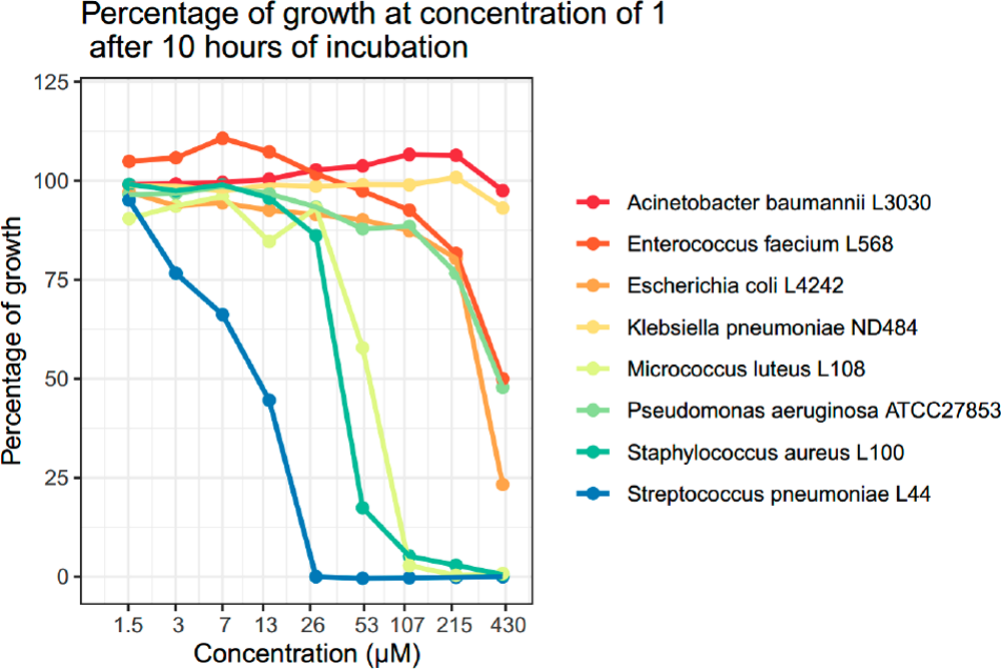
Antibacterial activity of compound **1**. Percentage of growth at 10 h in the presence of **1**. *E. coli* L4242 is a *ΔtolC* derivative of strain M1522. Note log scale on the *x* axis.

The aim of this study was to find novel antimicrobials using a bioactivity-independent approach. Indeed, compound **1** was found to suppress growth of *Streptococcus pneumoniae* and of *Staphylococcus aureus* with MICs of 12 and 43 μM, respectively (Figure 3). Minimal activity was also observed against *Micrococcus luteus* (68 μM), *Escherichia coli ΔtolC* (291 μM), *Pseudomonas aeruginosa*, and *Enterococcus faecium* (428 μM). Compound **1** was not cytotoxic to CaCo-2 and HEK cells (IC_50_’s of 154 and >220 μM at 24 h for CaCo-2 and HEK cells, respectively; Figure S25).

Bioactivity of the putative precursors streptophenazines A and F has been addressed in previous studies on different targets.^13,15,18,19^

### Biosynthetic Gene Cluster

Once **1** and **2** were known to belong to the streptophenazine class, we readily identified the BGC responsible for their formation from a draft genome of strain ID63040 with antiSMASH^22^ version 6.0 equipped with the MIBiG comparison tool.^23^

**Figure 4:**
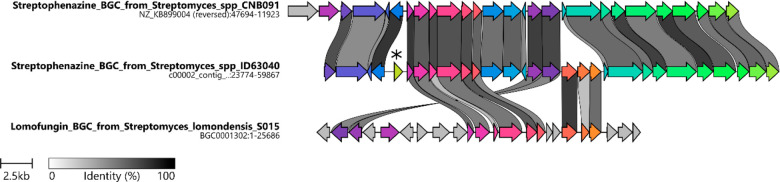
Comparison of streptophenazine BGC from strain ID63040 (middle) with reference streptophenazine BGC (top) from strain CNB091 and with lomofungin BGC from *Streptomyces lomondensis* S015 (bottom). The colors indicate similar genes. The asterisk marks a regulatory gene that is not found in reference clusters.

Figure 4 presents a comparison between the BGC in strain ID63040 and two reference clusters that contain sets of similar genes: one for streptophenazines, from the marine *Streptomyces* sp. CNB-091,^13^ and one for lomofungin, from *Streptomyces lomondensis* S015.^24,25^ The two streptophenazine BGCs are highly syntenic, sharing genes in similar order and orientation, apart from two insertions in the ID63040 BGC: a three-gene cassette, which is however present in the lomofungin BGC; and a regulator (yellow gene marked with an asterisk in Figure 4; see below). The three-gene cassette, which encodes for *S*-adenosylmethionine (SAM) synthetase, PfkB domain protein, and methylenetetrahydrofolate reductase, is likely involved in the cofactor regeneration for the SAM-dependent methyltransferase. A methyltransferase is present in all three BGCs of Figure 4 and is likely responsible for installing the methyl ester(s) on these molecules.

The regulatory gene embedded in the ID63040 BGC (locus tag ctg1_5) precedes the six-gene cassette responsible for synthesis of the phenazine core. While a homologue of this gene is present in the strain CNB-091, it is instead located four genes downstream from the left end of the CNB-091 streptophenazine BGC, in a closely linked BGC for the biosynthesis of mayamycin, a structurally unrelated aromatic polyketide (Figure S26). Interestingly, two precedents exist of (strepto)phenazine BGCs prioritized for heterologous expression, yet production was achieved only if the clusters were refactored by inserting strong promoters in front of the phenazine gene cassette,^13,26^ the exact same position where the regulatory gene naturally lies in the ID63040 BGC. This regulator shows 68% identity to JadR1, an activator of the jadomycin BGC and member of the OmpR family, referred to as “atypical response regulators”.^27^ Interestingly, JadR1 has been shown to respond to late products in the jadomycin pathway.^28^ Thus, a possibility exists that ctg1_5 could be responsible for constitutive production of streptophenazines in strain ID63040.

We then wondered about the co-occurrence of ctg1_5 in streptophenazine BGCs in genomic sequences. We searched the antiSMASH-DB^28^ with KnownClusterBlast for BGCs matching both the phenazine and the PKS portion of the streptophenazine BGC BGC0002010 and detected four BGCs containing both the phenazine gene cassette and the PKS portion, indicative of a streptophenazine BGC, out of a data set of 561 *Streptomycetales* genomes. In only one case was a ctg1_5 homologue embedded in the streptophenazine BGC, while in three other cases it was associated with a closely linked mayamycin BGC (Figure S26). Without associated metabolomics data, we can only speculate that the strain carrying the ctg1_5 homologue within the streptophenazine BGC might not require refactoring for streptophenazine production.

During their extensive analysis of streptophenazine metabolites from the native host and after introducing a refactored BGC into *Streptomyces coelicolor* M1146, Bauman et al. observed streptophenazines A, F, and G as main congeners.^13^ Upon close inspection of their data, we found that the strain carrying the refactored BGC also produced small amounts of compounds with *m*/*z* of 584.2413 and 570.2228 [M + H]^+^, which are consistent with **1** and **2**, respectively. Indeed, when the fragmentation patterns of the metabolites in both data sets were analyzed with the MASST tool,^29^ both **1** and **2** were found in data sets associated with results published by Bauman et al.^13^ with a cosine score of 0.78 and 0.74 for *m*/*z* 570.1783 and 584.1971, respectively (Table S3).

It should be noted that Bauman et al. characterized *N*-formyl-glycine esters at the C-1′ OH of streptophenazines A and B as minor components of the complex. Thus, position C-1′ can undergo different modifications requiring different mechanisms in different strains: esterification of the hydroxy group as reported by Bauman et al. and thioether formation in the present case. Interestingly, Bauman et al. observed that knocking out the gene Spz15 impaired the formation of the *N*-formyl-glycinated product. However, we doubt that the Spz15 homologue present in the ID63040 BGC is making the thioether bond observed in **1** and **2** (see below).

### Congener Abundance Dynamics

Since compounds **1** and **2** are structurally related to streptophenazines F and A, respectively, we investigated whether the latter compounds could also be detected. Indeed, by close inspection of extracts from strain ID63040, we observed *m*/*z* [M + H]^+^ values of 439 (F) and 425 (A), which showed similar fragmentation patterns to **1** and **2**, respectively, and superimposable UV spectra (Figure S1). Using the Moldiscovery workflow on GNPS, we confirmed the presence of streptophenazines A and F, as well as congeners B, C, D, E, G, K, N, and O (data not shown). Of note, none of these molecules clustered into the same molecular family as **1** and **2** in molecular networks calculated on GNPS.

**Figure 5:**
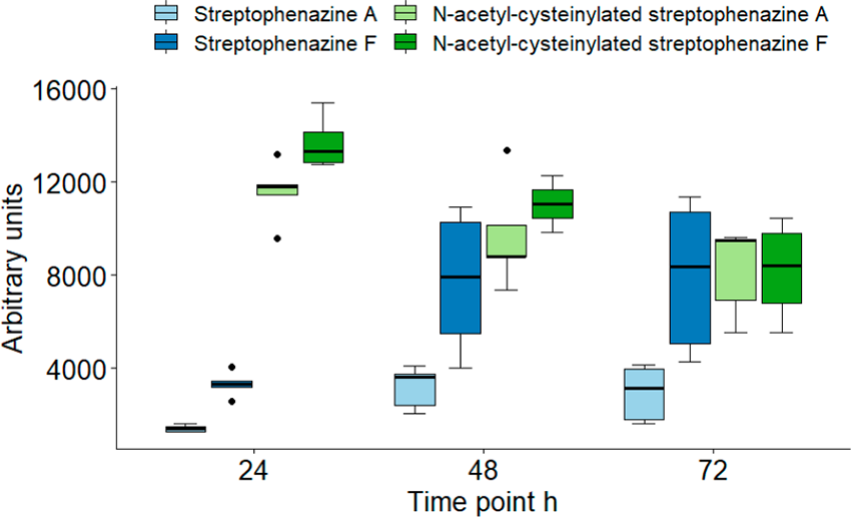
Abundances of 1′-(*N*-acetycysteinyl)-1′-deoxystreptophenazines A and F (greens) and streptophenazines A and F (blues) at 24, 48, and 72 h. Results are expressed as the average of five biological replicates run in parallel (500 mL of M8 medium).

To determine the relative abundances of the different streptophenazine congeners in the mycelium extracts, we used UV measurements. Unlike the intensities of the *m*/*z* peaks that may depend strongly on ionization efficiency, dimerization, charges per molecule, and adduct formation, UV measurements enable reliable quantification of target compounds that share a common chromophore, i.e., the phenazine ring. In Figure 5, we report the peak heights at 252 nm associated with each *m*/*z* value. Compounds **1** and **2** were the prevalent congeners at 24 h, when A and F are hardly detectable. Only at 72 h did streptophenazine F become comparable in concentration to **1** and **2**. Thus, during exponential growth *Streptomyces* sp. ID63040 produced **1** and **2** as the main components of the streptophenazine complex, while streptophenazines A and F became significant metabolites only when the strain enters a stationary phase. To make sure this was not a peculiarity of our test system, we explored congener ratios in four different media: INA5, G1/0, SV2, and M8.^4^ In all media where we observed production, *N*-acetyl-cysteinylated variants were the most abundant forms (Figure S27), in line with our observations for production medium of compounds **1** and **2**.

### On the Possible Role of Streptophenazines

Phenazine is a moiety present in many known chemicals, both of synthetic and natural origin.^30^ The properties of the phenazine scaffold depend on pH, making it fit for a variety of tasks within and between microbial cells, ranging from carrying out redox functions and modulating gene expression to acting as signaling molecules and inhibiting growth of competitors.^31^ Various mechanisms of action have been associated with phenazine antibiotics; for example, myxin has been shown to intercalate with DNA, lomofungin inhibits RNA synthesis, and pyocyanine provokes oxidative damage.^32^ The best-known producers of naturally occurring phenazines are pseudomonads and streptomycetes,^32^ the latter group featuring also their own special group of phenazine-derived metabolites named streptophenazines, which consist of an aliphatic tail added to the phenazine core by a polyketide synthase.

**Chart 1:**
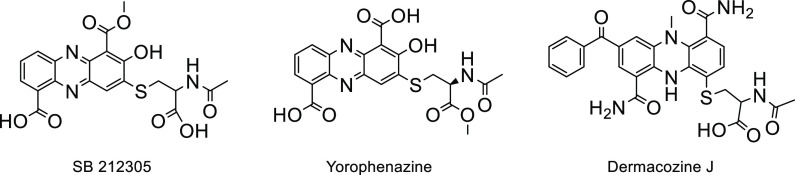
Structures of Yorophenazine, SB212305, and Dermacozine J^33−35^

Previously, other phenazine natural products have been described carrying an *N*-acetyl-cysteine moiety attached through a thioether bridge to the aromatic core: examples are yorophenazine,^33^ SB 212305,^34^ and dermacozine J^35^ (Chart 1). Of note, all these molecules are devoid of the polyketide tail.

An *N*-acetyl-cysteine moiety has also been reported in several nonphenazine natural products,^36−41^ and it has been hypothesized to result from mycothiol addition, a compound present in many actinobacteria, where it is used to maintain cellular redox balance, to serve as a pool of stabilized cysteine, and, in analogy to glutathione, to detoxify xenobiotics.^42−44^ The mechanism of detoxification involves *S*-conjugation to the target molecule, followed by cleavage of the amide bond linking *N*-acetyl-cysteine to glucosamine in mycothiol. There are precedents for metabolites that have been shown or hypothesized to gain the *N*-acetyl-cysteine moiety from mycothiol: one such example is nanaomycin,^45^ for which the authors described both the *N*-acetyl-cysteinylated and mycothiolated forms of the pyronaphthoquinone core. Thus, it is tempting to speculate that **1** and **2** are formed by such a mechanism.

While only few reports exist on the *in vivo* kinetics of *N*-acetyl-cysteine adduct(s) formation, this appears to be a slow process: for example, the mercapturic acid of granactin A appeared in cultures of *S. violaceruber* TÜ7 only after 9 days, coinciding with the disappearance of native granactin A.^43^ By contrast, the cysteinylated products were the prevalent form during active growth of ID63040. A possible explanation of our findings might be that in strain ID63040 streptophenazines participate in an important redox function and, in doing so, are transformed into a toxic form that is readily neutralized by conjugation with mycothiol. It could be that as exponential growth slows or ceases, the role of streptophenazines in redox function decreases and congeners devoid of the *N*-acetyl-cysteine moiety (i.e., the true end product of the biosynthetic pathway) start to accumulate. If this hypothesis is correct, it would imply that streptophenazines in strain ID63040 play a role akin to primary metabolites and hence their constitutive production through a BGC-embedded regulator. However, further experimental work would be necessary to validate these propositions. Of note, Price-Whelan et al.^31^ have previously stated that “phenazines blur the line between primary and secondary metabolism”.

## Conclusions

In summary, we have described two *N*-acetyl-cysteinylated streptophenazines with antibacterial activity identified through a metabolomics-first approach. Thus, it is possible to discover metabolites with antibacterial activity through a bioactivity-independent approach, even from a relatively small number of *Streptomyces* strains, a genus that has been intensively exploited for secondary metabolites, yet found to be by far the richest in terms of BGC diversity.^47^ The prioritization criteria described herein represent just one such strategy, but we are confident that additional new chemistry has been overlooked by our prioritization criteria, leaving room for further studies.

Our results also point out the importance of the host in shaping the final metabolite profile. While it is well known that many BGCs are not expressed under laboratory conditions,^46,47^ BGC-specified metabolites can also undergo further spontaneous or enzymatic modifications as they are exposed to the cellular milieu.^45,48,49^ The crosstalk between primary and secondary metabolism pathways has recently yielded new members even for one of the oldest known families of antibiotics,^49^ underlining the somewhat arbitrary distinction between the two.

## Experimental Section

### General Experimental Procedures

ECD spectra were obtained on a J-815 spectropolarimeter (JASCO). IR spectra were recorded with a Varian 670-IR. ^1^H and ^13^C 1D and 2D NMR spectra (COSY, TOCSY, NOESY, HSQC, HMBC) were measured in DMSO-*d*_6_ and acetone-*d*_6_ at the indicated temperature using a Bruker Advance II 300 MHz spectrometer (^1^H NMR 300 MHz, ^13^C NMR 75 MHz). The 1D ^13^C NMR spectrum for compound **1** was measured in DMSO-*d*_6_ at 300–350 K using a Bruker Avance III 600 spectrometer (^1^H NMR 600 MHz, ^13^C NMR 150 MHz). Chemical shifts were referenced relative to the corresponding signals (δ_H_ 2.05/δ_C_ 29.8 for acetone-*d*_6_; δ_H_ 2.50/δ_C_ 39.50 for DMSO-*d*_6_).

LC-MS/MS analyses were performed on a Dionex UltiMate 3000 HPLC system coupled with an LCQ Fleet (Thermo Scientific) mass spectrometer equipped with an electrospray interface (ESI) and a tridimensional ion trap. The column was an Atlantis T3 C-18 5 μm × 4.6 mm × 50 mm maintained at 40 °C at a flow rate of 0.8 mL/min. Phase A was 0.05% trifluoroacetic acid (TFA); phase B was 100% MeCN. The elution was executed with a 14 min multistep program that consisted of 10%, 10%, 95%, 95%, 10%, and 10% phase B at 0, 1, 7, 12, 12.5, and 14 min, respectively. UV–vis signals (190–600 nm) were acquired using the diode array detector. The *m*/*z* range was 110–2000, and the ESI conditions were as follows: spray voltage of 3500 V, capillary temperature of 275 °C, sheath gas flow rate at 35 units, and auxiliary gas flow rate at 15 units.

High-resolution MS spectra were recorded at Unitech OMICs (University of Milano, Italy) using a Dionex Ultimate 3000 HPLC system coupled with a Triple TOF 6600 (Sciex) equipped with an ESI source. The experiments were carried out using the same column and eluting system as described above. The ESI parameters were the following: curtain gas 25 units, ion spray voltage floating 5500 V, temperature 50 °C, ion source gas 1 10 units, ion source gas 2 0 units, declustering potential 80 V, syringe flow rate 10 μL/min, accumulation time 1 s.

### Production and Purification of Streptophenazines

For production of **1**, 1.5 mL of the −80 °C stock culture was inoculated into 100 mL of AF medium (dextrose monohydrate 20 g L^–1^, yeast extract 2 g L^–1^, soybean meal 8 g L^–1^, NaCl 1 g L^–1^, and CaCO_3_ 3 g L^–1^, pH 7.3, prior to autoclaving^4^) in two 300 mL baffled flasks and grown for 3 days at 30 °C at 200 rpm. AF culture (30 mL) was transferred into five 2 L flasks with four baffles containing 500 mL of M8 medium (dextrose monohydrate 10 g L^–1^, yeast extract 2 g L^–1^, meat extract 4 g L^–1^, soluble starch 20 g L^–1^, casein 4 g L^–1^, and CaCO_3_ 3 g L^–1^, pH 7.2, prior to autoclaving).^4^ The culture was harvested at 72 h and filtered through Whatman paper, and the mycelium was extracted with 430 mL of 100% EtOH by shaking for 1 h at 30 °C. The extract (ca. 500 mL) was filtered through Whatman paper and through a 0.2 μm syringe filter and dried with a rotary evaporator, yielding 19.2 g of solids.

For production of **2**, 0.75 mL of frozen culture was inoculated into 50 mL baffled Erlenmeyer flasks containing 10 mL of medium AF. After 72 h at 30 °C on an orbital shaker at 200 rpm, 5 mL of the culture was transferred into each of six baffled 500 mL Erlenmeyer flasks containing 150 mL of fresh AF medium. After another 72 h at 30 °C, 500 mL of culture was used to inoculate a 5 L fermenter (New Brunswick, BioFlo/CelliGen 115) containing 4.5 L of medium M8 supplemented with 0.5 mL/L polypropylene glycol (Sigma) as antifoaming agent. The fermenter was operated under the following parameters: agitation 400 rpm, temperature 30 °C, air flow 5 mL/min. The culture was harvested at 48 h and filtered through Whatman paper, and the resulting 1.2 L of mycelium was extracted with 1 L of 100% EtOH by shaking overnight at room temperature. The extract (ca. 1.3 L) was filtered three times through Whatman paper and dried with a rotary evaporator, yielding 36.3 g of solids.

After drying, the residues were dissolved in 50% dimethylformamide (DMF) and fractionated separately using a TeledyneISCO CombiFlash MPLC mounted with a Biotage SNAP Ultra C_18_ 25 μm 60 g column with detection at 252 nm. Phases A and B were 0.05% TFA and MeCN, respectively. The gradient used was 5%, 5%, 60%, 95%, and 95% phase B at 0, 5, 10, 30, and 35 min, respectively. Throughout the purification procedure, the presence of **1** and **2** was monitored by LC-MS. For structure elucidation, we used a 3.8 mg sample of **1** and a 5.2 mg sample of **2**. Typical yields of **1** and **2** were 2–5 mg from 1 L of culture.

#### 1′-(*N*-acetycysteinyl)-1′-deoxystreptophenazine A (**1**):

yellowish-brown amorphous solid; UV–vis (MeCN, *c* 0.01 mg/mL) λ_max_ (log ε) 206 (4.8), 253 (4,2), 366 (3.5) nm; ECD (1.9 mmol/L, MeOH) λ_max_ (Δε) −0.5 (265), + 2.5 (245), −1 (230) nm; IR (neat) (ν_max_) 3300, 2950, 2850, 1750, 1650, 1530, 1450, 1250, 1200, 1100 cm^–1^; ECD and IR spectra see Figures S25 and S2; NMR data, Table 1; HRESIMS *m*/*z* 584.2430 [M + H]^+^ (calcd for C_30_H_35_N_3_O_7_S, 584.2430).

#### 1′-(*N*-acetycysteinyl)-1′-deoxystreptophenazine F (**2**):

yellowish-brown amorphous solid; UV–vis (MeCN, *c* 0.01 mg/mL) λ_max_ (log ε) 206 (4.8), 253 (4.2), 366 (3.5) nm; NMR data, Table 1; HRESIMS *m*/*z* 570.2277 [M + H]^+^ (calcd for C_29_H_37_N_3_O_7_S, 570.2274).

### Derivatization of **1** and **2**

The number of free carboxyl groups in **1** and **2** was established by methylation and amidation reactions. For derivatization reactions, we dried aliquots of the MPLC fraction, each containing 0.5 mg of a mixture of **1** and **2**. All the reactions were followed by HPLC-LRMS. For methylation, compounds **1** and **2** were dissolved in 50 μL of MeOH and treated with 2 μL of H_2_SO_4_ at room temperature. For amidation, compounds **1** and **2** were dissolved in 100 μL of dry DMF and treated with 2 μL of ethylene diamine diluted 1:50 in dry DMF in the presence of a few crystals of HATU (1-[bis(dimethylamino)methylene]-1*H*-1,2,3-triazolo[4,5-*b*]pyridinium 3-oxide hexafluorophosphate).

The number of methyl esters was confirmed by basic hydrolysis. Compounds **1** and **2** were treated with 2 N NaOH at room temperature, and the reaction mixture was analyzed by LC-MS prior to and at 5 min, 1 h, and 24 h after NaOH addition.

### Bioassays

The antibacterial activity of compound **1** was determined against strains from the NAICONS collection of bacterial pathogens as follows: 90 μL of a 1 × 10^5^ CFU/mL bacterial suspension in the appropriate medium was dispensed into each well of a 96-well plate containing 10 μL of compound **1**, dissolved at 428 μM in 2.5% DMSO, and serially diluted 1:2 with 2.5% DMSO. The medium was cation-adjusted MHB for all strains except for *S. pneumoniae*, for which THB was used. Plates were incubated in a Synergy 2 (Biotek) microplate reader with readings at 595 nm registered every hour.

For cytotoxicity assays, HEK 293 cells and CaCo-2 cells were cultured in DMEM/F-12 and DMEM (Gibco), respectively, supplemented with 10% fetal calf serum (FCS) and a 1% penicillin–streptomycin mixture (Gibco) at 37 °C and 5% CO_2_. Cells were seeded in 96-well plates at a concentration of 1 × 10^5^ cells per well, and the outer wells were filled with PBS to avoid disturbances due to evaporation. After 24 h of incubation, cells were confluent. Twofold dilutions of the compounds were made in a range of 214–6.7 μM, in exposure medium. The exposure medium consisted of DMEM without phenol red (Gibco), without supplementation with FCS or antibiotics. The growth medium from the plates was aspirated and replaced with 100 μL of exposure medium containing different concentrations of the compounds. Three wells were used as a medium control, three as vehicle control (1% DMSO), and three as positive control for toxicity (20% DMSO). After 24 h of exposure, cytotoxicity was measured using the Alamar Blue assay (Thermo Fisher Scientific) by adding 10 μL of reagent per well, incubating 1 h at 37 °C 5% CO_2_, and measuring fluorescence (λ_EX_ = 540 nm, λ_EM_ = 590 nm) on a SpectraMax M5 (Molecular Devices). The IC_50_ was calculated using GraphPhad Prism 9 using a nonlinear regression analysis: log(inhibitor) vs response – variable slope (four parameters) without constraints.

### Bioinformatic Analyses

From a draft genome sequence of *Streptomyces* strain ID63040, obtained using both Illumina short read sequencing and Oxford Nanopore Technologies long read sequencing and consisting of 11 contigs with a total length of 8 915 670 bp (K.V. and V.W, unpublished), BGCs were identified using the antiSMASH version 6.0 online tool.^23^ To search for the occurrence of homologues of the regulator ctg1_5 in streptophenazine BGCs, we searched the antiSMASH-DB^28^ with KnownClusterBlast for BGCs matching both the phenazine and the PKS portion of the streptophenazine BGC BGC0002010. BGC alignments were visualized with Clinker.^50^ The streptophenazine BGC from *Streptomyces* strain ID63040 was deposited to GenBank with accession no. OL619055.

The 16S rRNA gene sequence (GenBank accession no. OL423644) was determined and analyzed as described.^51^

## Supporting Information Available

LRESIMS and MSMS spectra of hydrolyzed, derivatized, and native **1** and **2**; IR spectrum of **1**; ECD spectrum of **1** in MeOH; ^1^H NMR, COSY, TOCSY, HSQC, and HMBC of **1** in acetone-*d*_6_ at 300 K; ^1^H NMR of **1** in DMSO-*d*_6_ with stepwise temperature increase from 300 to 350 K; COSY, TOCSY, NOESY of **1** in DMSO-*d*_6_ at 350 K; COSY, TOCSY, and NOESY of **1** in DMSO-*d*_6_ at 350 K; 1D ^13^C NMR of **1** in DMSO-*d*_6_ at 340 K; HSQC and HMBC of **1** in DMSO-*d*_6_ at 350 K; ^1^H NMR, COSY, and HSQC of **2** in acetone- *d*_6_ at 300 K; cytotoxicity data of compound **1** on CaCo-2 and HEK cell lines; comparison of streptophenazine BGC regions of strain ID63040 and of other *Streptomyces* streptophenazine BGCs from the antiSMASH database; abundances of **1** and **2** and streptophenazines A and F in four media at three time points; calculated molecular formulas for parent mass, selected fragments, and neutral losses of compounds **1** and **2**; strains reported to produce streptophenazines and BGC sequence availability; hits for *N*-acetyl-cysteinylated streptophenazines **1** and **2** in public databases (PDF)
Raw NMR data files (ZIP)
