# Supplementary material for: N-Acetyl-Cysteinylated Streptophenazines from Streptomyces
Source: J Nat Prod. 2022 Apr 15;85(5):1239–47. doi: 10.1021/acs.jnatprod.1c01123 (PMC9150181; doi:10.1021/acs.jnatprod.1c01123)
Supplement: Supplementary file 1 — np1c01123_si_001.pdf [file np1c01123_si_001.pdf]

N-Acetyl-Cysteinylated Streptophenazines from *Streptomyces*

Authors: Kristiina Vind[*,†,‡], Sonia Maffioli[†], Blanca Fernandez Ciruelos[‡], Valentin Waschulin[§], Cristina Brunati[†], Matteo Simone[†], Margherita Sosio[†], Stefano Donadio[*,†]*

[†] NAICONS Srl, 20139 Milan, Italy

[‡] Host-Microbe Interactomics Group, Wageningen University, 6708 WD Wageningen, The Netherlands

[§] School of Life Sciences, University of Warwick, Coventry, United Kingdom

*kristiina.vind@wur.nl

*sdonadio@naicons.com

Table of Contents

Figure S1. Fragmentations of **1** and **2** and their putative precursors....2
Figure S2. IR spectrum of **1**....3
Figure S3. Alkaline hydrolysis of **1** and **2**. 4
Figure S4. Derivatization of **1** and **2**. 5
Figure S5. 1H-NMR of **1** in acetone-d6 at 300K....6
Figure S6. COSY of **1** in acetone-d6 at 300K....7
Figure S7. TOCSY of **1** in acetone-d6 at 300K. 8
Figure S8. HSQC of **1** in acetone-d6 at 300K. 9
Figure S9. HMBC of **1** in acetone-d6 at 300K. 10
Figure S10. 1H-NMR of **1** in dmso-d6 with stepwise temperature increase from 300K to 350K....11
Figure S11. 1H-NMR of **1** in dmso-d6 with stepwise temperature increase from 300K to 350K. Selected signals. 12
Figure S12. COSY of **1** in dmso-d6 at 350K. 13
Figure S13. TOCSY of **1** in dmso-d6 at 350K. 14
Figure S14. NOESY of **1** in dmso-d6 at 350K. 15
Figure S15. COSY, TOCSY and NOESY of **1** in dmso-d6 at 350K. 16
Figure S16. 1D 13C-NMR of **1** in dmso-d6 at 340K. 17
Figure S17. HSQC of **1** in DMSO-d6 at 350K. 18
Figure S18. HMBC of **1** in DMSO-d6 at 350K. 19
Figure S19. 1H-NMR of **2** in acetone-d6 at 300K. 20
Figure S20. COSY of **2** in acetone-d6 at 300K. 21
Figure S21. HSQC of **2** in acetone-d6 at 300K. 22
Figure S22. HSQC and HMBC for **2** and **1** in acetone-d6 at 300K: aromatic portion 23
Figure S23. HSQC and HMBC for **2** and **1** in acetone-d6 at 300K: central portion 24
Figure S24. HSQC and HMBC for **2** and **1** in acetone-d6 at 300K: aliphatic portion 25
Figure S25. ECD spectrum of **1** in MeOH. 26
Figure S26. Cytotoxicity of compound **1** on CaCo-2 and HEK cell lines. 27
Figure S27. Comparison of streptophenazine BGC regions of strain ID63040 and of other Streptomyces streptophenazine BGCs from the antiSMASH database....28
Figure S28. Abundances of **1** and **2** and streptophenazines A and F in four media (G1/0, INA5, M8 and SV2) at three timepoints (24, 48 and 72 h)....29
Table S1. Calculated molecular formulae for parent mass, selected fragments and neutral losses of compounds **1** and **2**. 30
Table S2. Strains reported to produce streptophenazines and BGC sequence availability. 31
Table S3. Hits for N-acetyl-cysteinylated streptophenazines **1** and **2** in public databases. 32

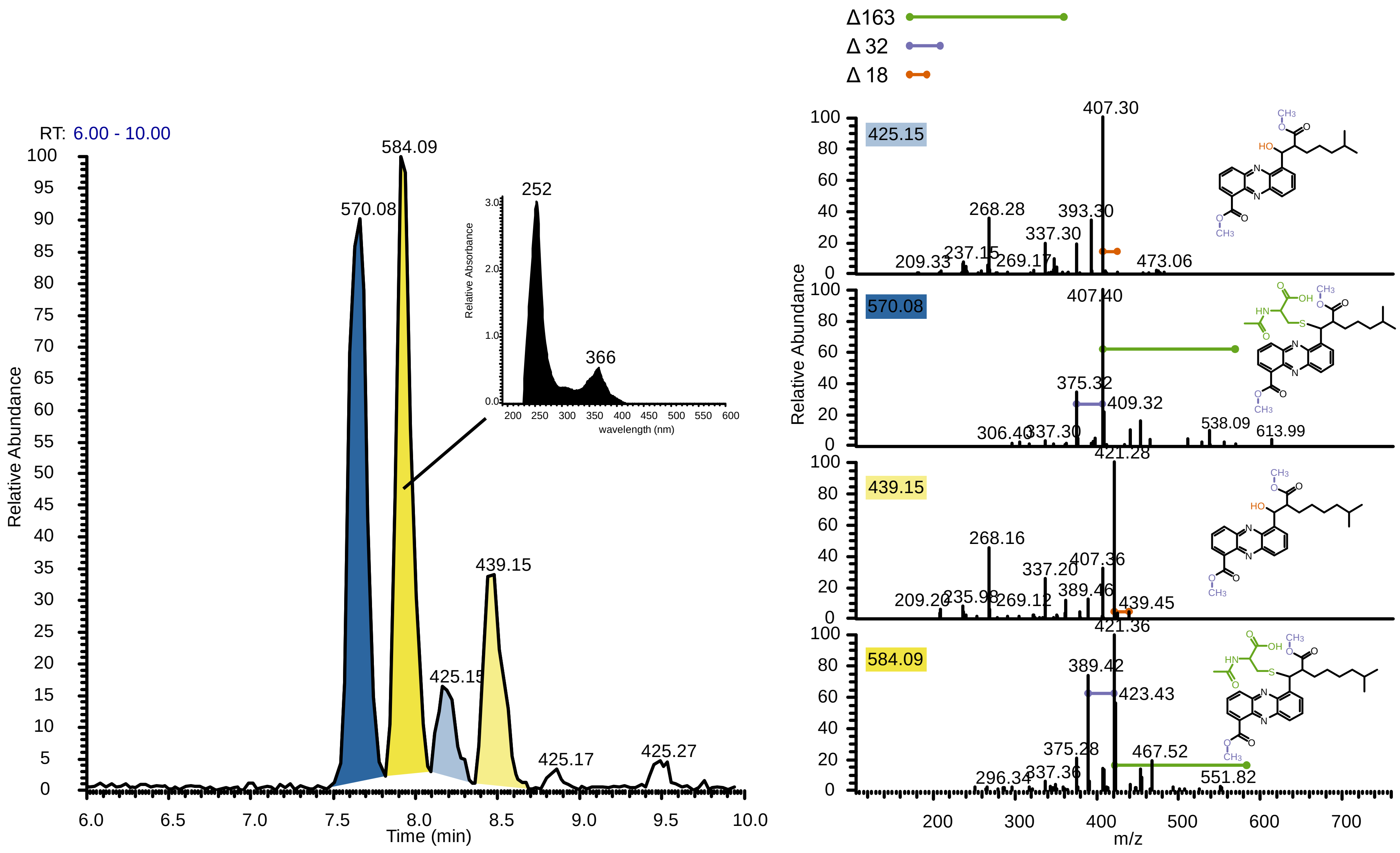

*Figure S1. Fragmentations of **1** and **2** and their putative precursors. Left: EIC of m/z [M + H]⁺ values 584.4, 570.4, 425.2 and 439.2., corresponding to **1** (yellow background) and **2** (blue background) or to their precursors (background in lighter shade). Embedded: UV-vis absorption spectrum corresponding to m/z 584.4 [M + H]⁺. Right: fragmentation patterns of **1**, **2** and their precursors. Horizontal bars indicate the size of neutral losses with corresponding part highlighted in structure with same color. Data was acquired with low resolution LC-MS instrument.*

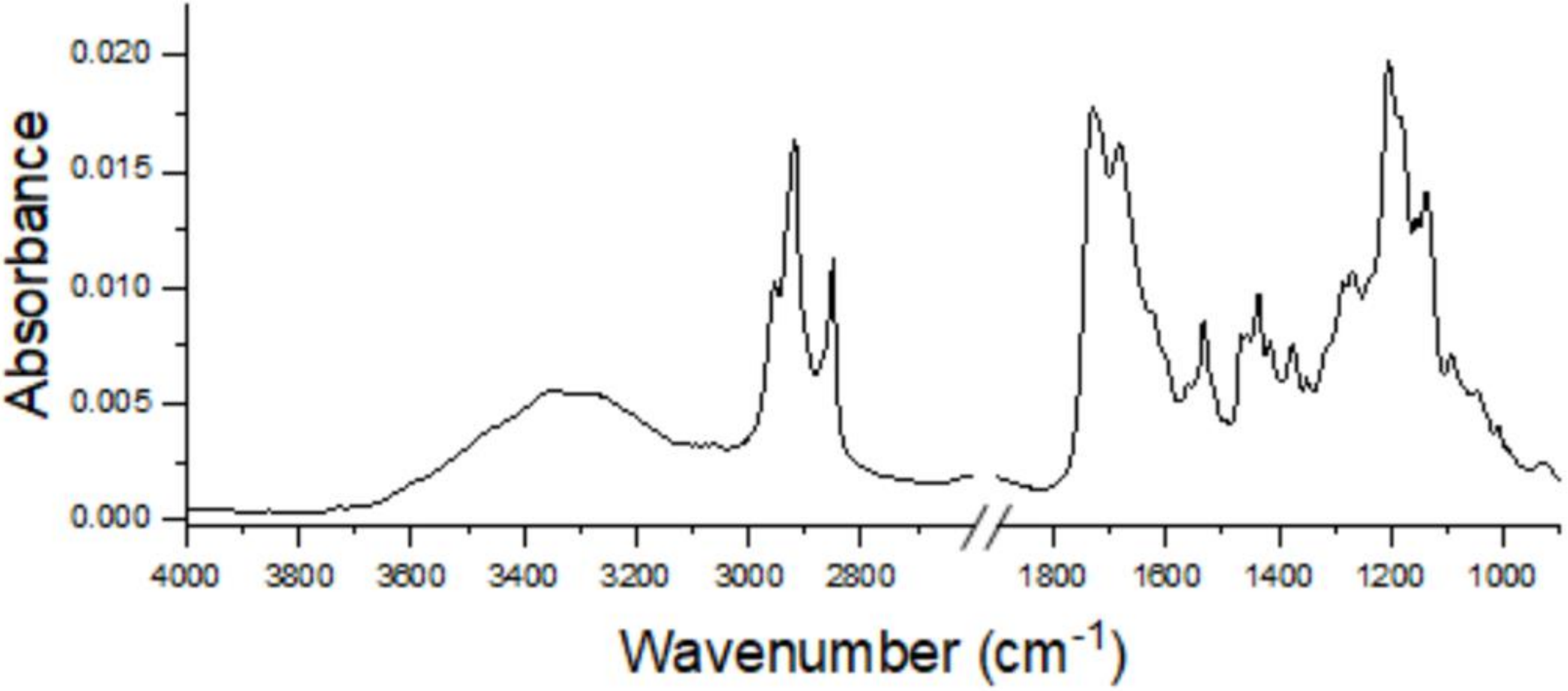

*Figure S2. IR spectrum of **1**. 2 µl of sample in MeOH was loaded on quarz support, solvent was evaporated, and the sample was scanned.*

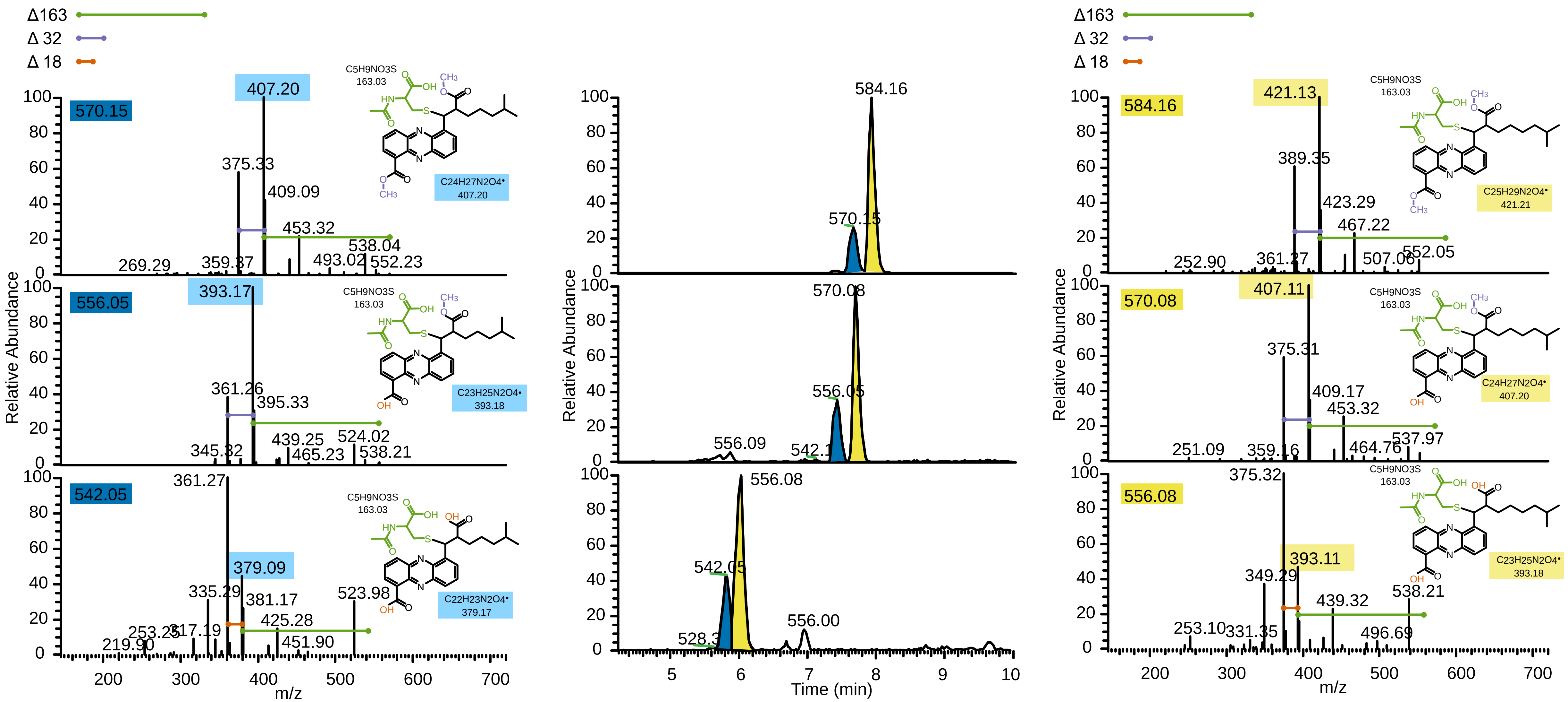

*Figure S3. Alkaline hydrolysis of **1** and **2**. Middle panel: Extracted Ion Chromatogram (EIC) of m/z [M + H]+ values 584.4, 570.4, 556.4, 542.4 and 528.4., corresponding to **1** (yellow background) and **2** (blue background) or their derivatives. From top to bottom: analyses before treatment, 5 minutes and 24 hours after treatment with 2N NaOH. Left panel: fragmentation patterns of **2** and its derivatives. Right panel: fragmentation patterns of **1** and its derivatives. The bars reflect the size of neutral loss on left and right panels: green corresponds to a neutral loss of 163 (N-acetylcysteine), purple to 32 (methyl ester) and orange to 18 (water). Data was acquired with low resolution LC-MS instrument. Note that the different hydrolytic behavior of methyl esters (green ink) was established by an NMR analysis (see text).*

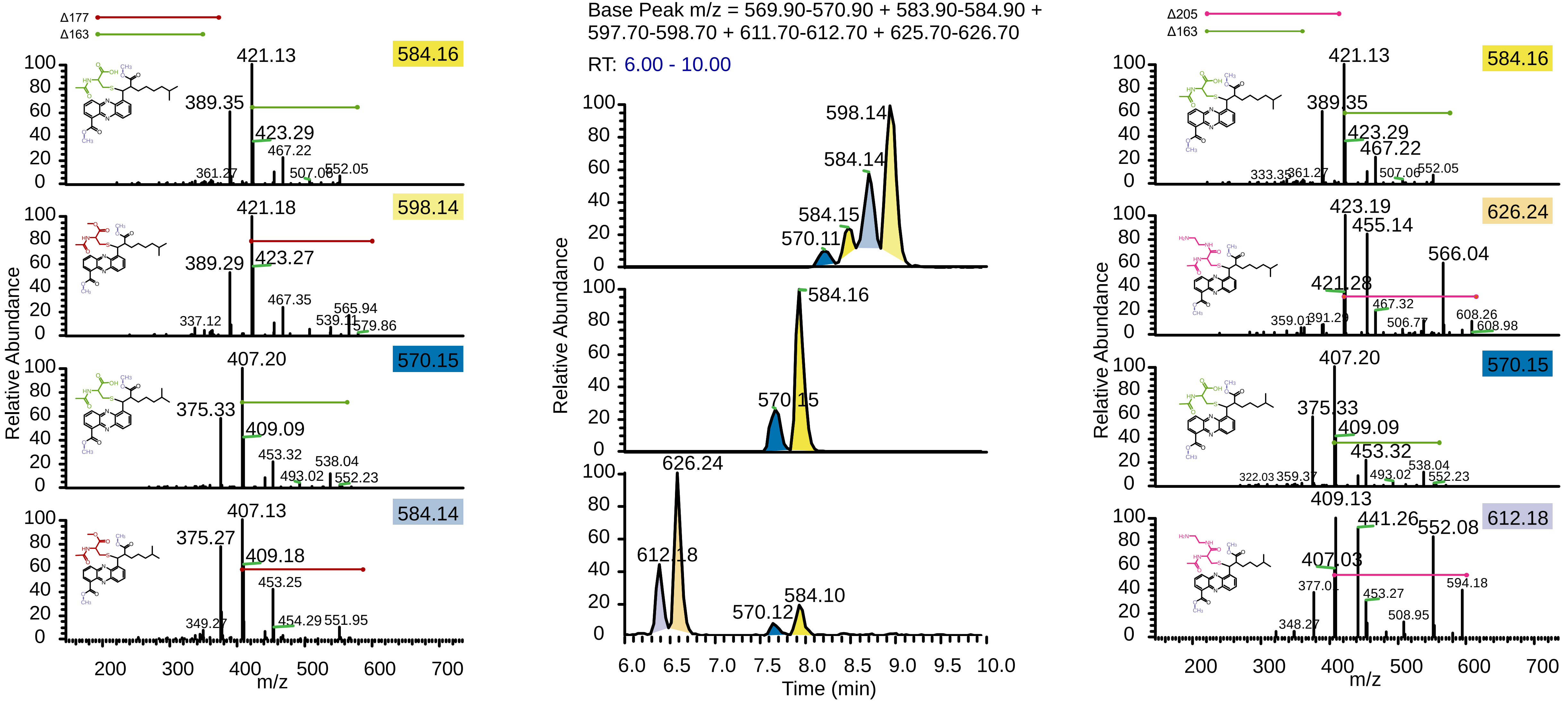

*Figure S4. Derivatization of **1** and **2.** Middle: EIC of m/z [M + H]$^+$ values 584.4, 570.4, 584.4, 598.2, 621.4 and 626.2, corresponding to **1** (yellow background) and **2** (blue background) or their derivatives. Left: MSMS patterns of native and methylated products. Right: MSMS patterns of native and amidated products. The bars reflect the size of neutral loss on left and right panels: green corresponds to a neutral loss of 163 (N-acetylcysteine), dark red to 177 (N-acetylcysteine + methyl) and pink to 205 (N-acetylcysteine + ethylenediamine). Data was acquired with low resolution LC-MS instrument.*

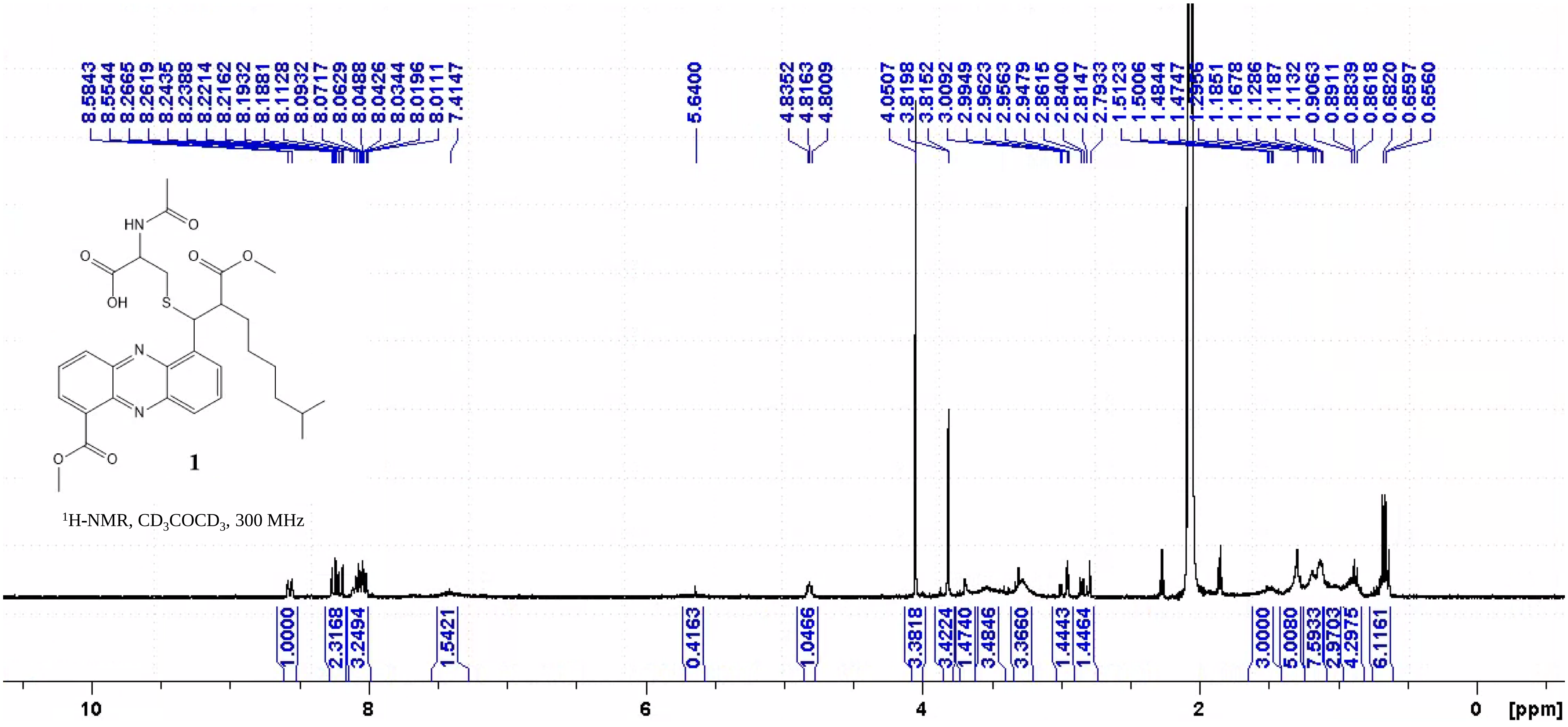

*Figure S5. ^{1}H-NMR of **1** in acetone-d_6 at 300K*

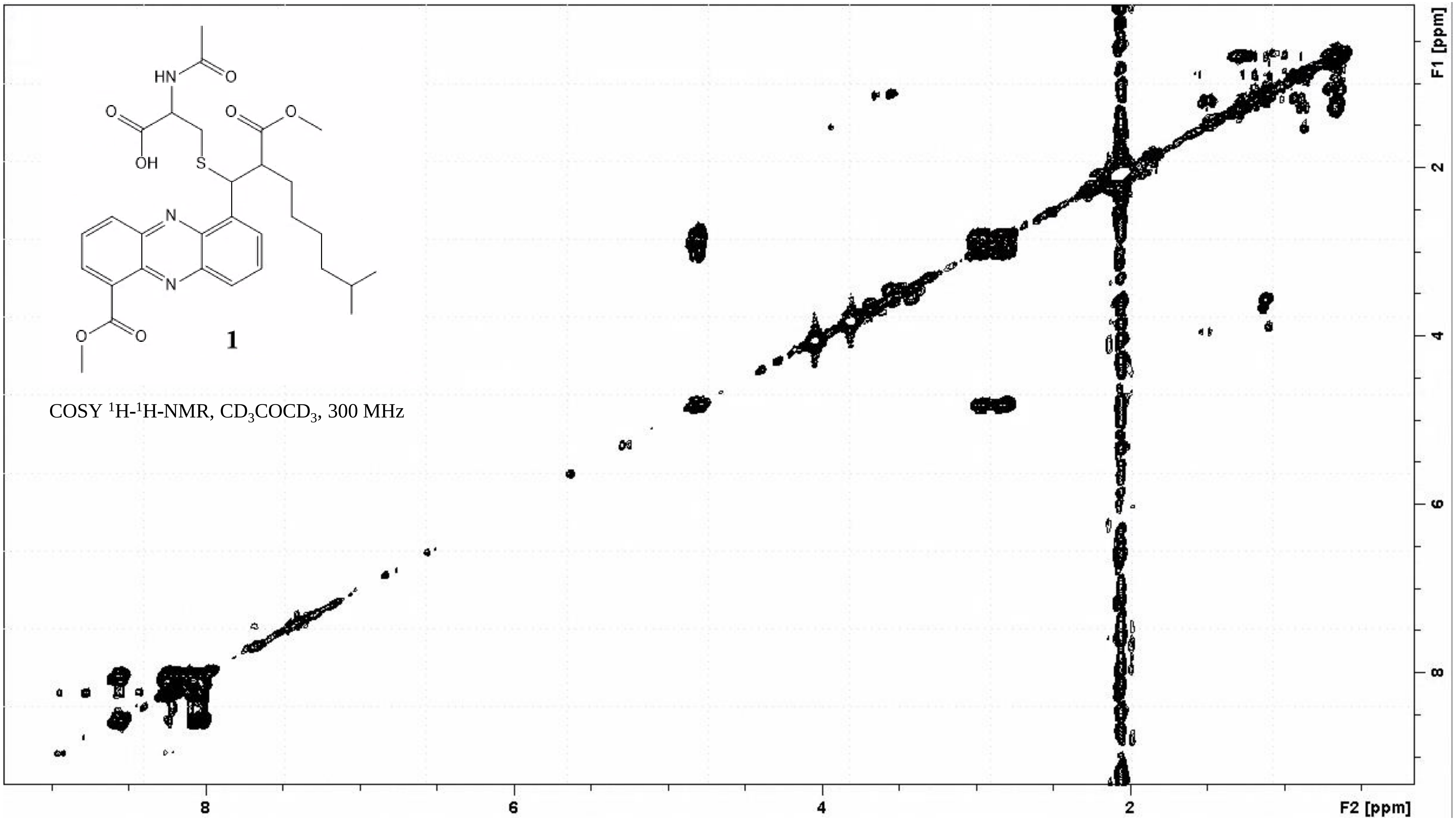

*Figure S6. COSY of **1** in acetone-d_6 at 300K*

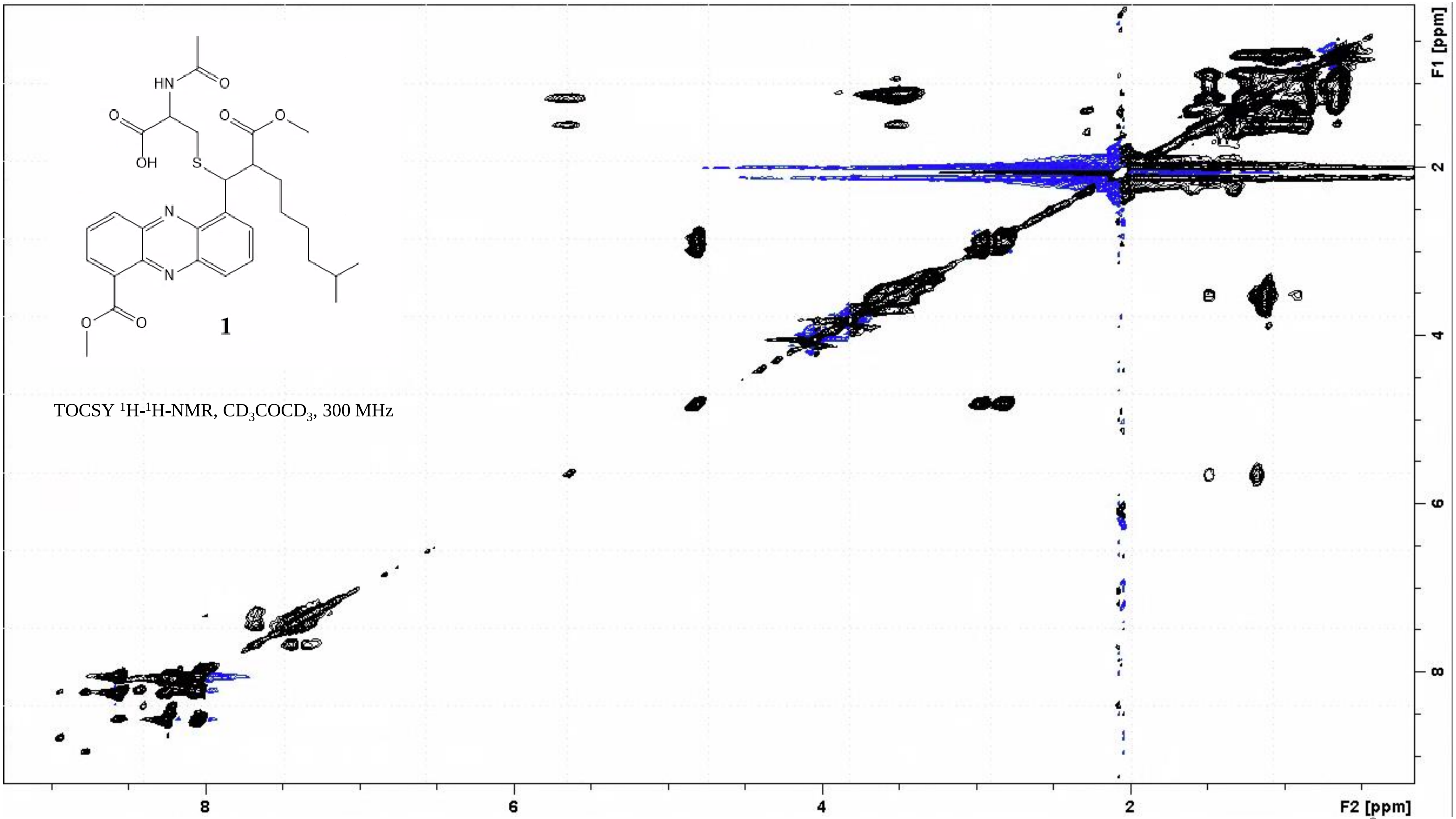

*Figure S7. TOCSY of **1** in acetone-d_6 at 300K*

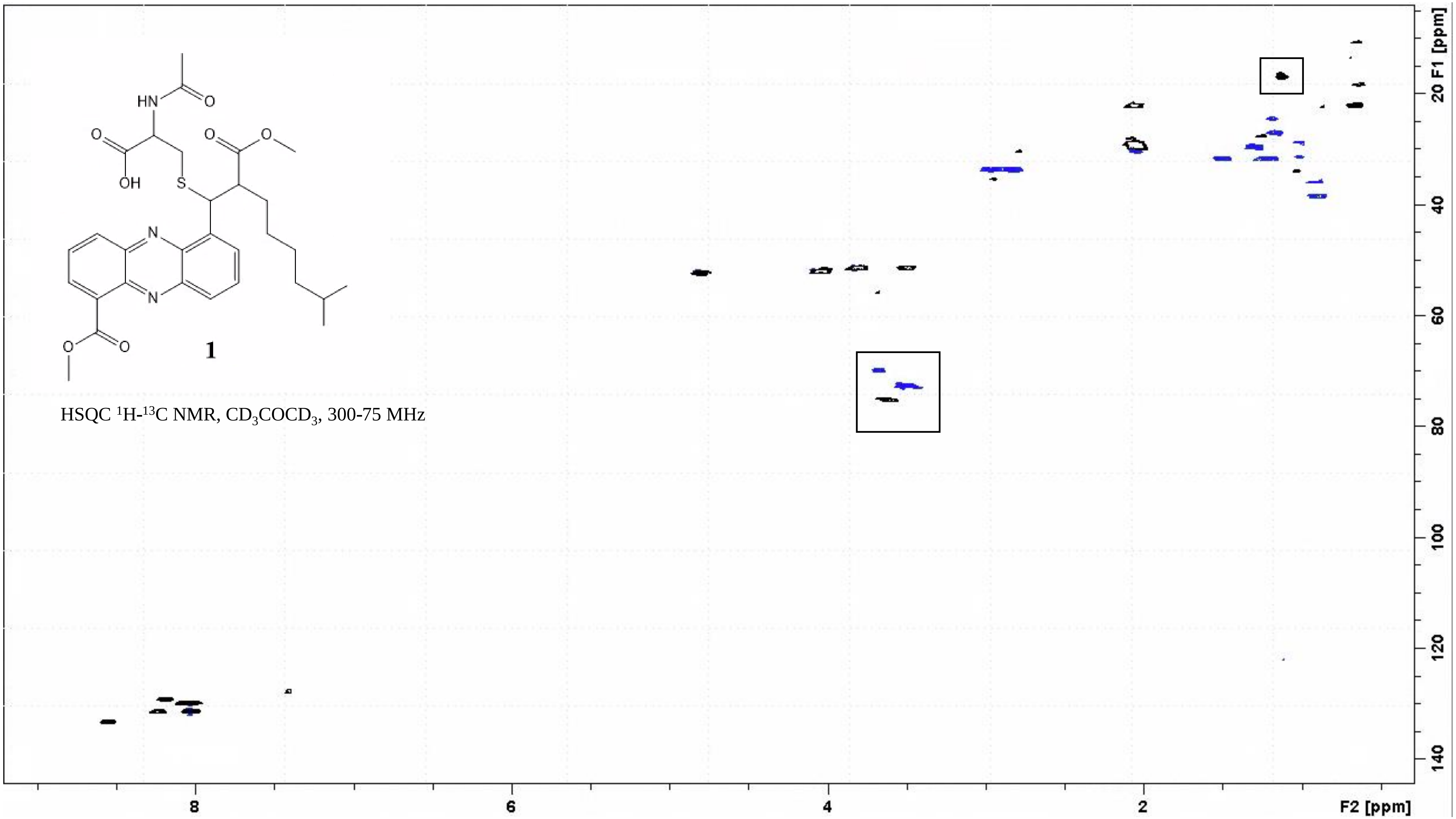

*Figure S8. HSQC of **1** in acetone-d_6 at 300K. Boxed: signals from unrelated impurities*

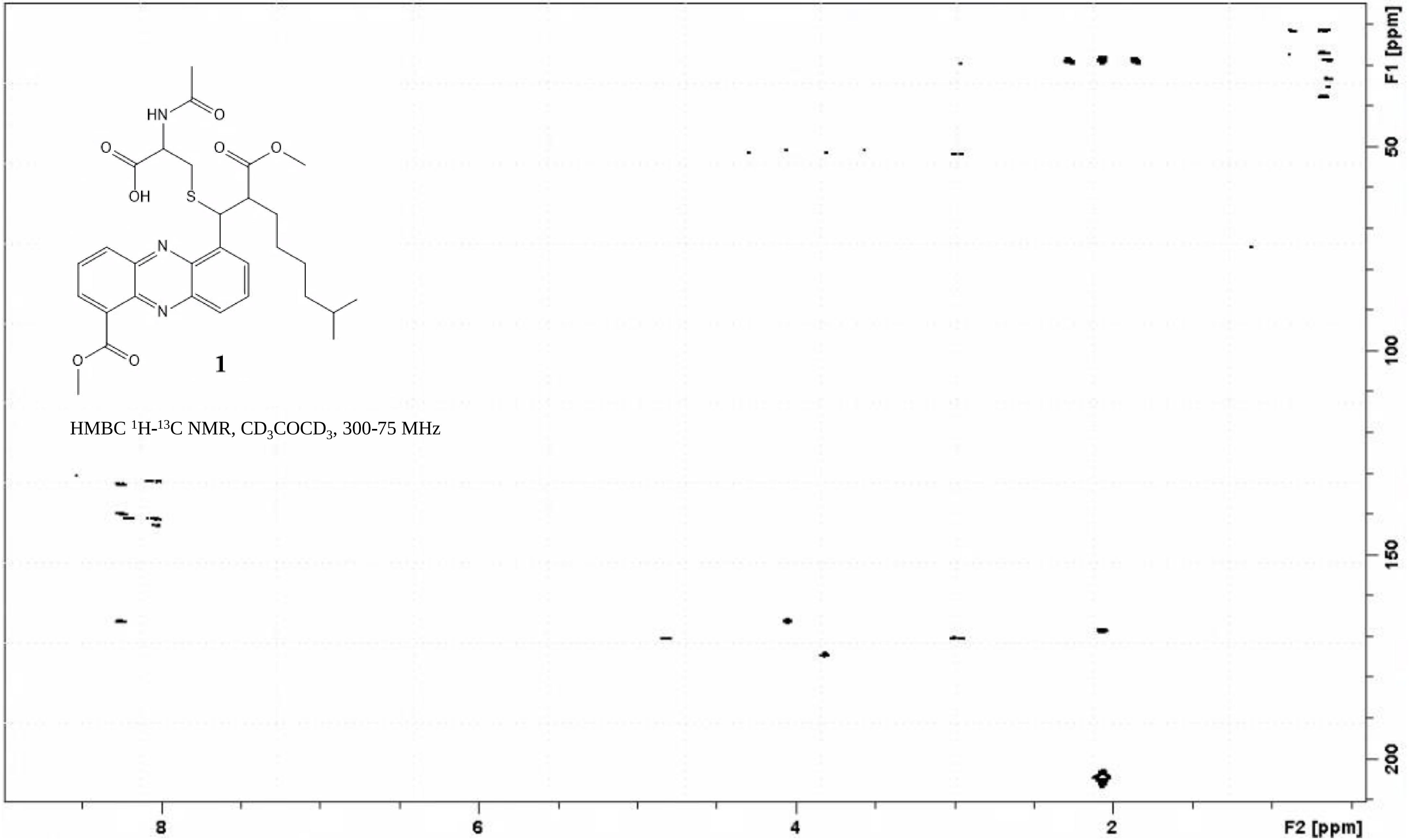

*Figure S9. HMBC of **1** in acetone-d_6 at 300K.*

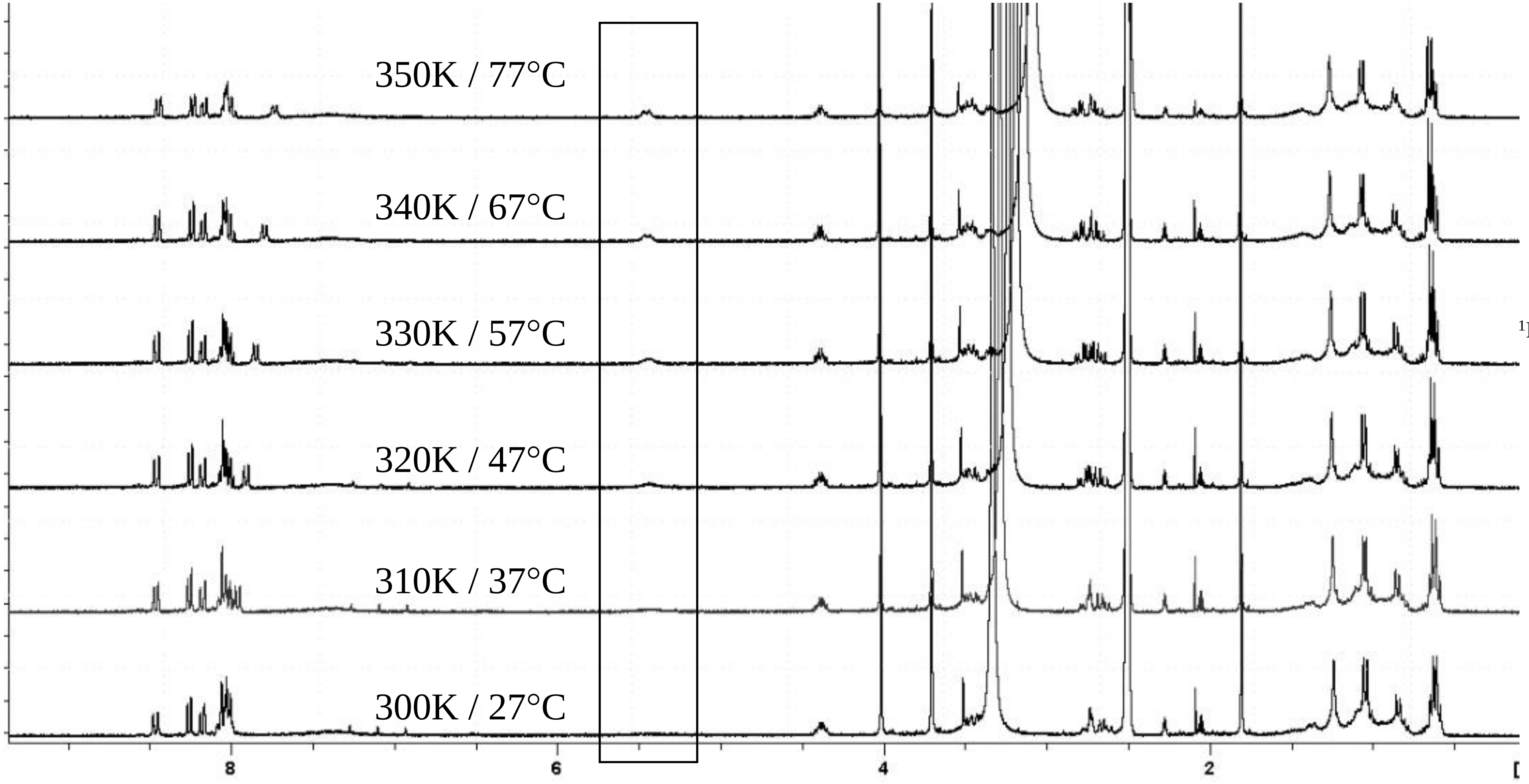

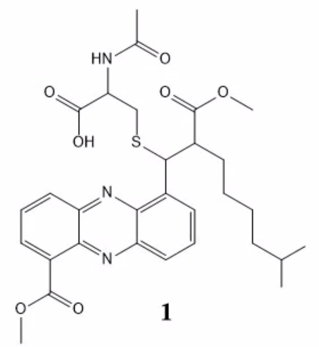

^{1}H-NMR, CD_3SOCD_3, 300 MHz

*Figure S10. ^{1}H-NMR of **1** in dmso-d_6 with stepwise temperature increase from 300K to 350K. Boxed: signal from H-1'*

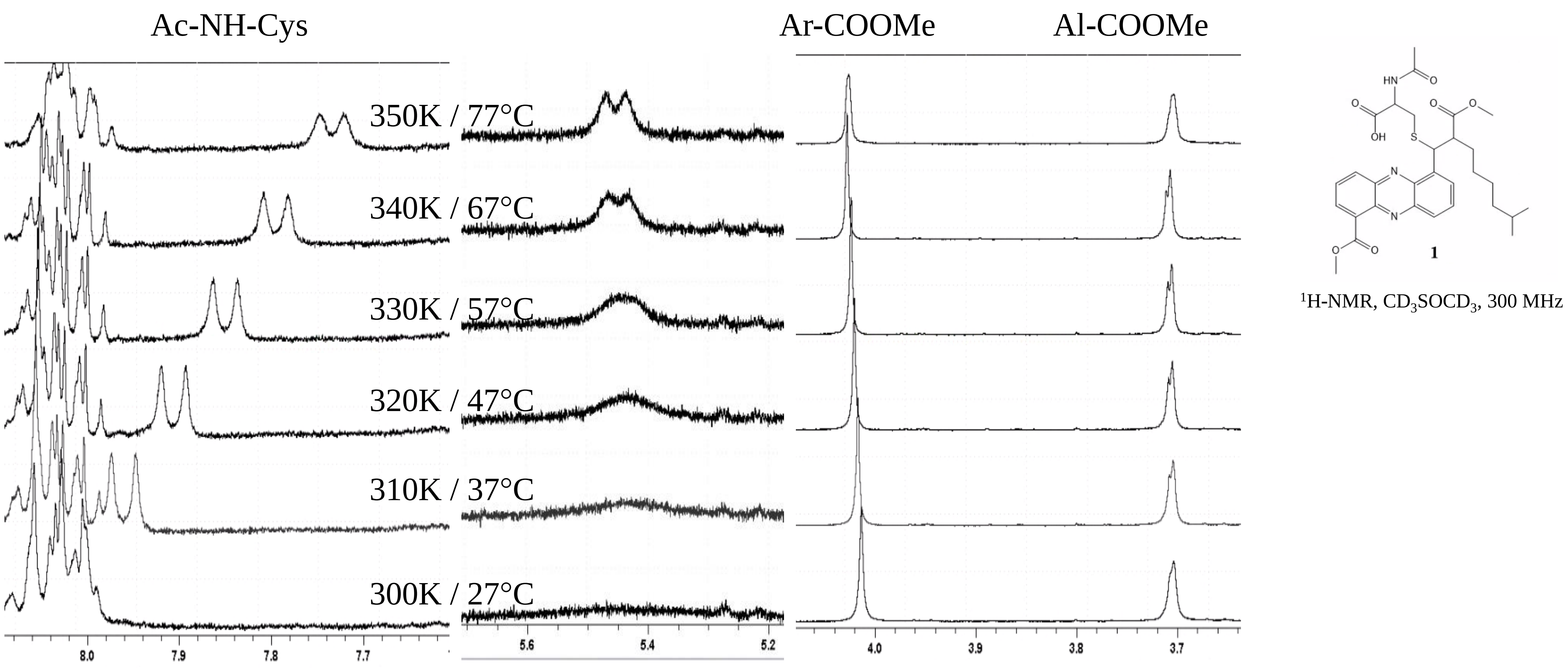

*Figure S11. ¹H-NMR of **1** in dmso-d_6 with stepwise temperature increase from 300K to 350K. Selected signals*

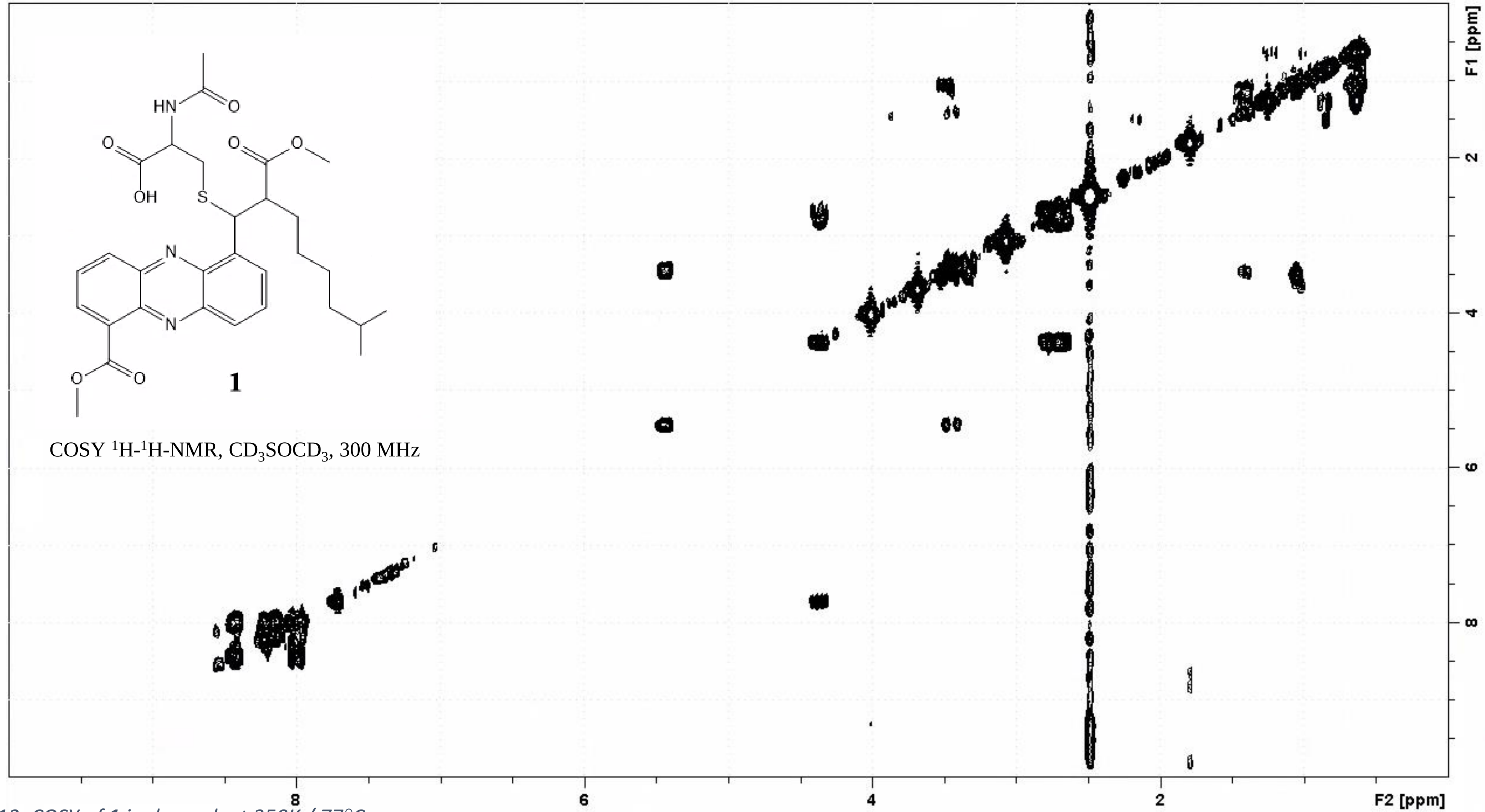

*Figure S12. COSY of **1** in dmso-d_6 at 350K / 77°C*

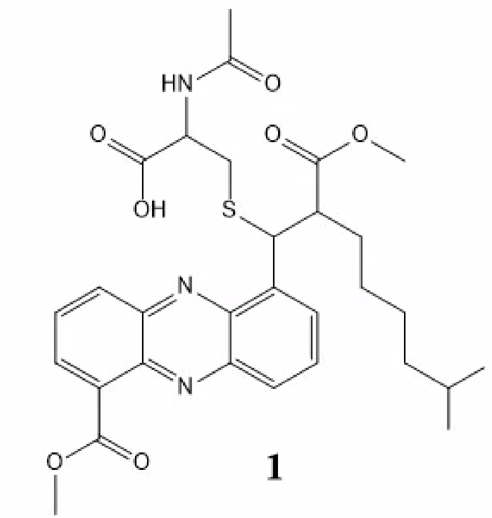

1

TOCSY ^{1}H-^{1}H-NMR, CD_3SOCD_3, 300 MHz

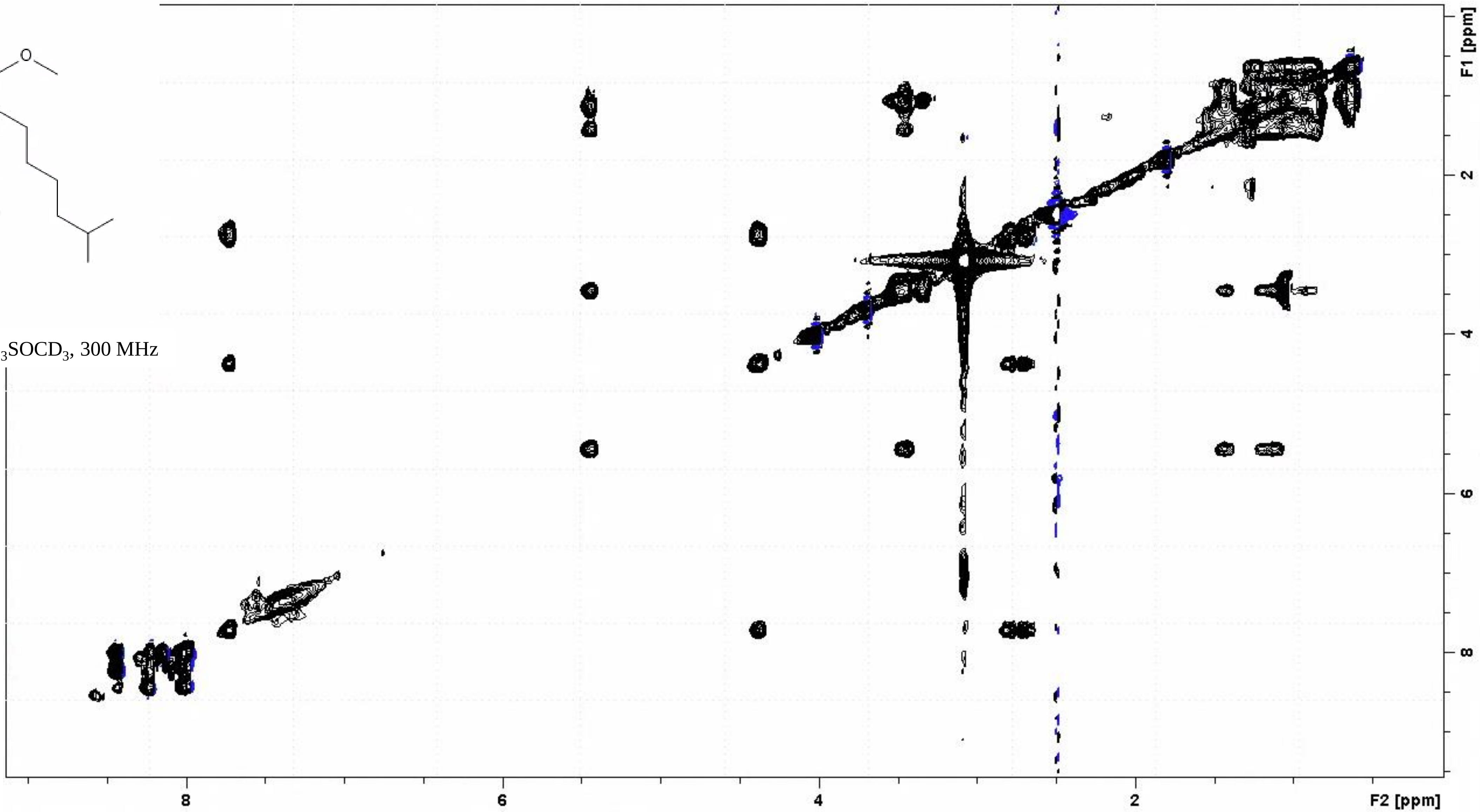

*Figure S13. TOCSY of **1** in dmso-d_6 at 350K / 77°C*

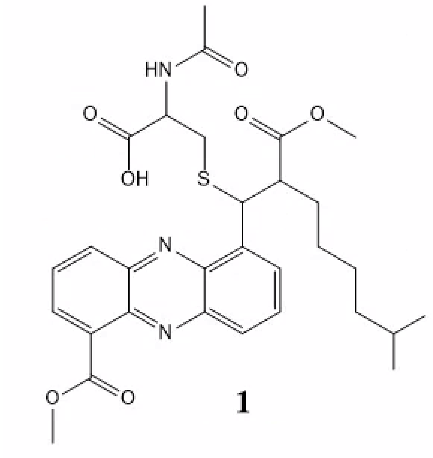

NOESY ^1H-^1H-NMR, CD_3SOCD_3, 300 MHz

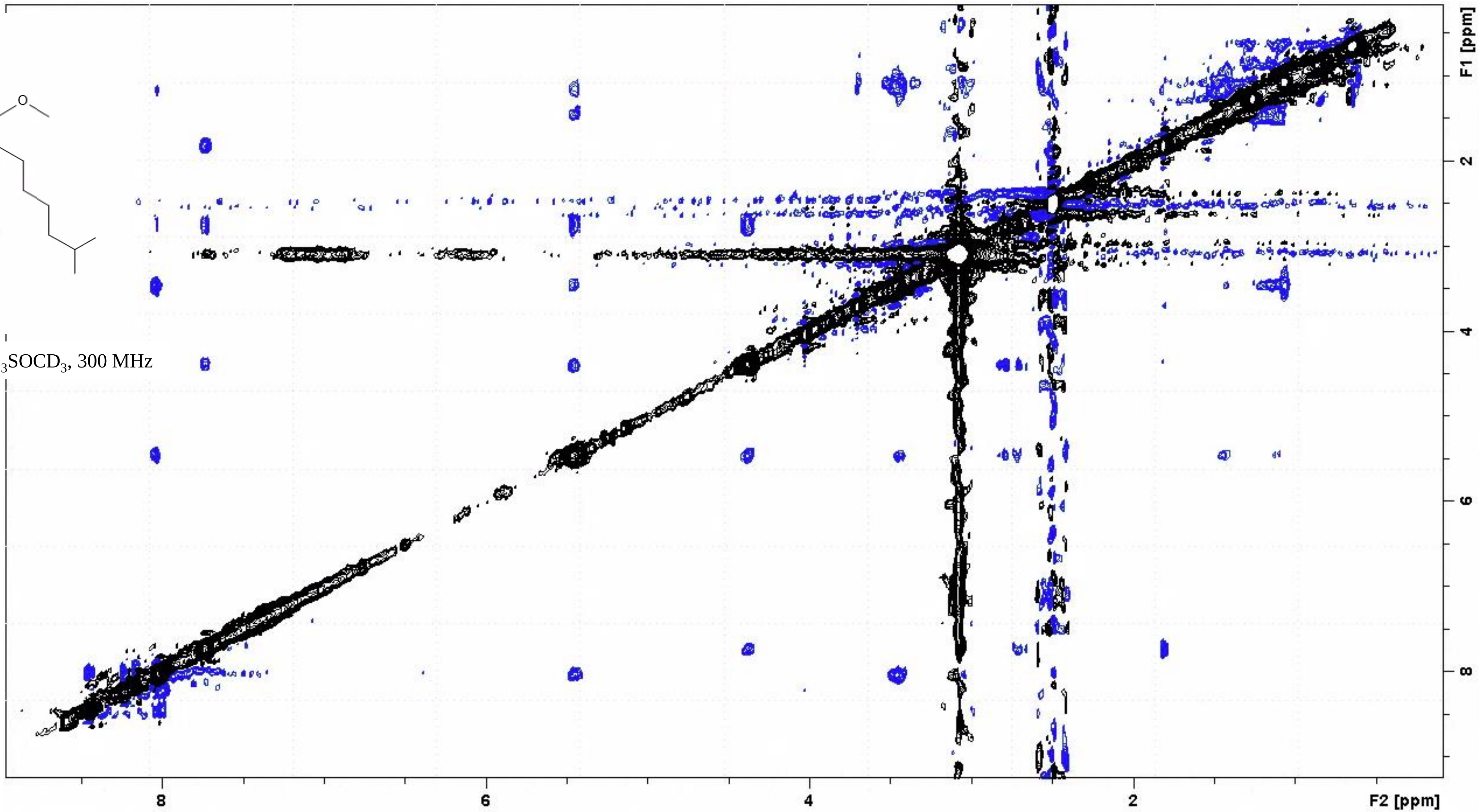

*Figure S14. NOESY of **1** in dmso-d_6 at 350K / 77°C*

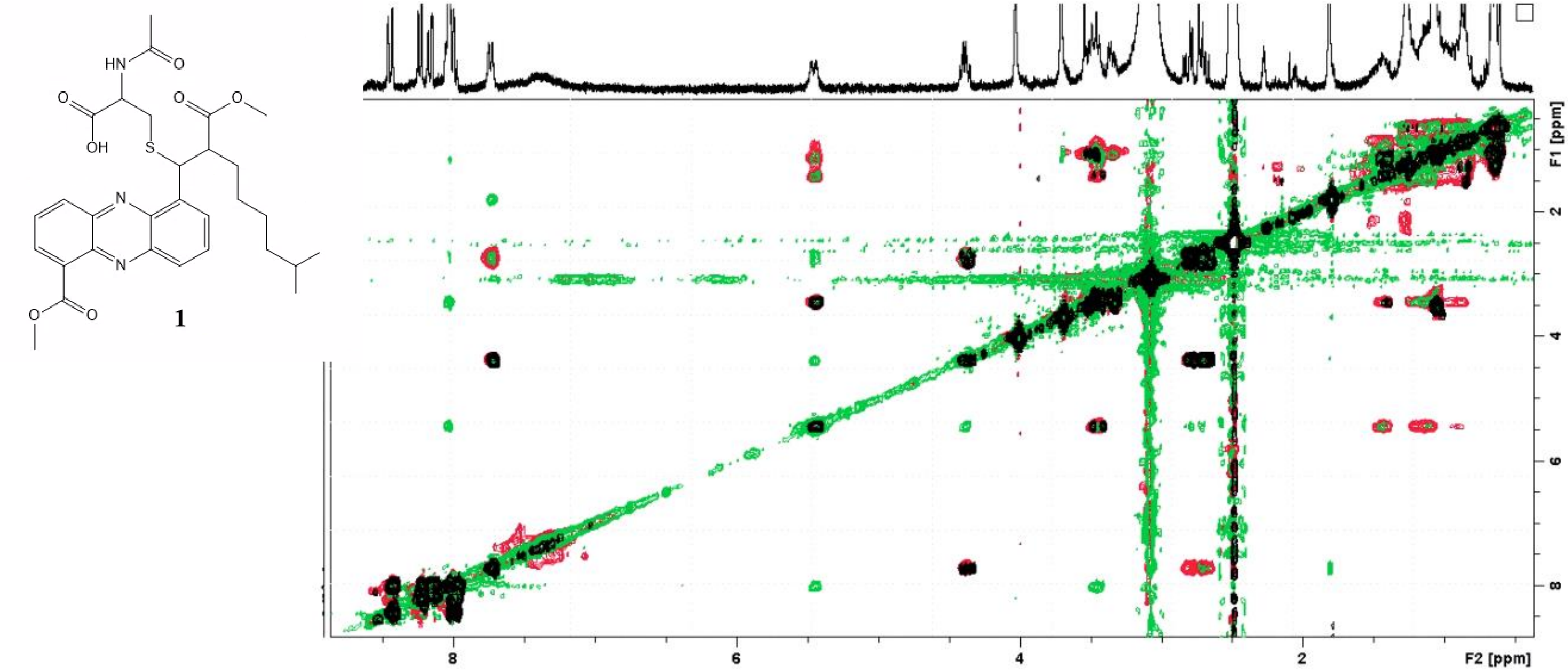

*Figure S15. COSY (black), TOCSY (red) and NOESY (green) of **1** in dmso-d$_6$ at 350K / 77°C*

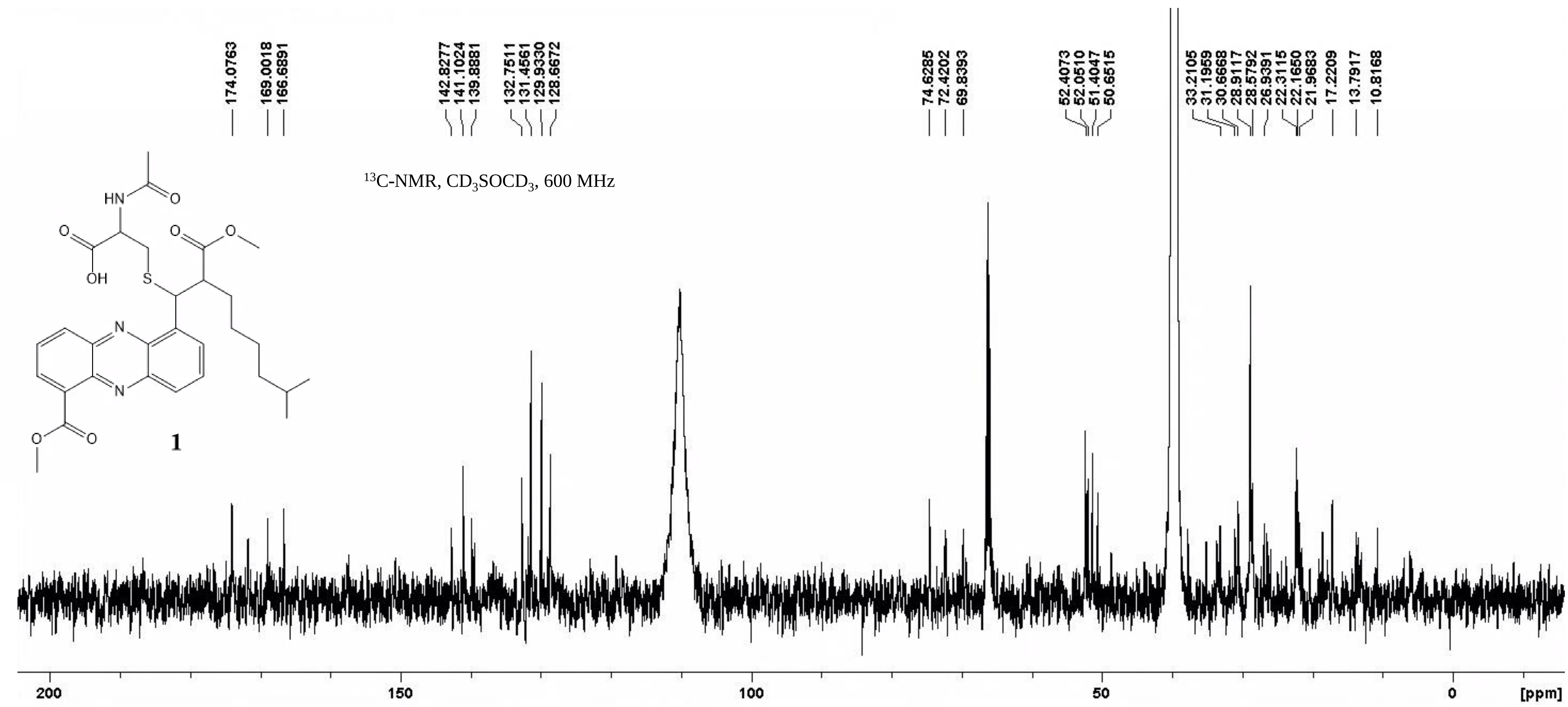

Figure S16. 1D ^{13}C-NMR of 1 in dmso-d6 at 340K / 67°C

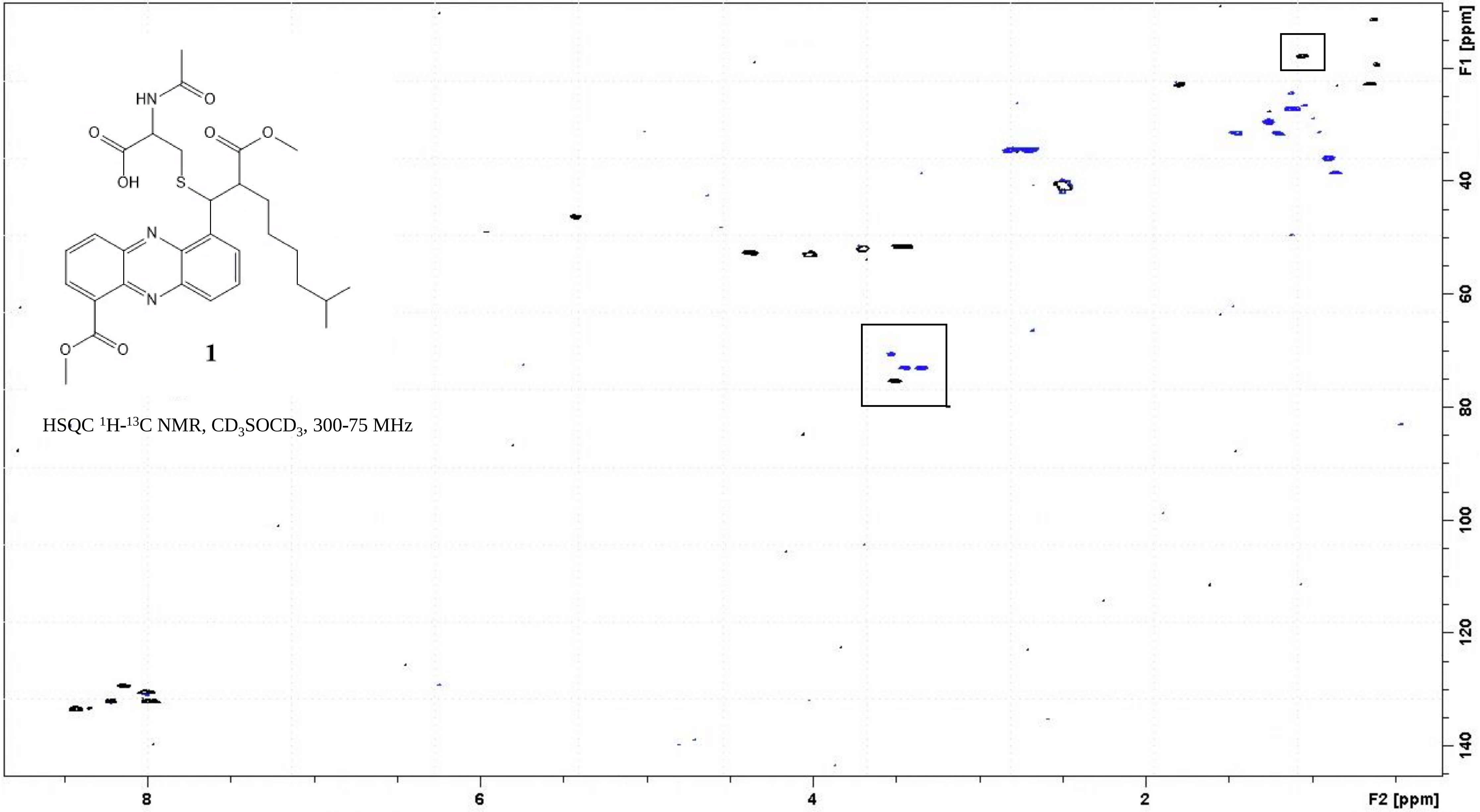

*Figure S17. HSQC of **1** in DMSO-d$_6$ at 350K / 77°C. Boxed: signals from unrelated impurities*

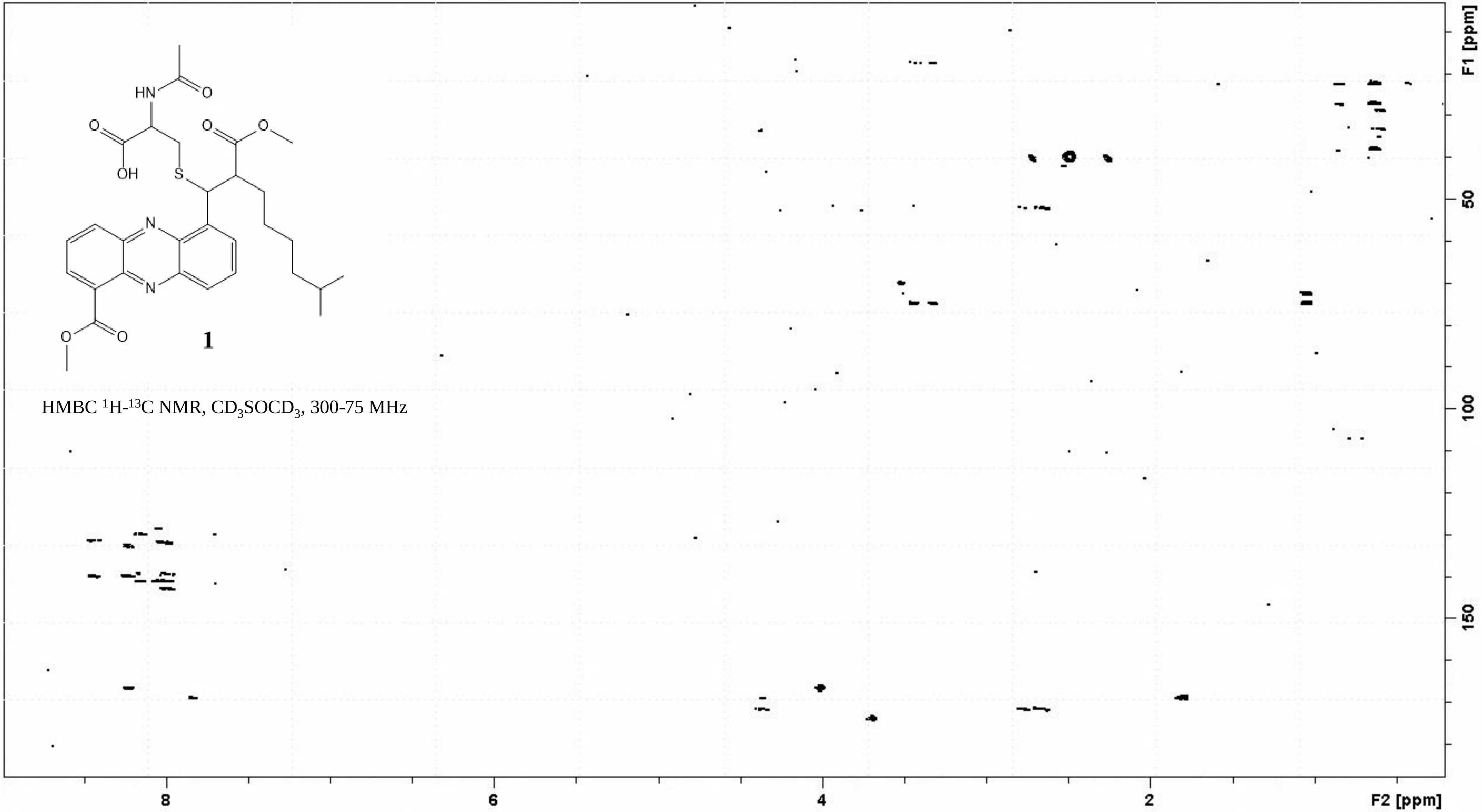

*Figure S18. HMBC of **1** in DMSO-d_6 at 350K / 77°C.*

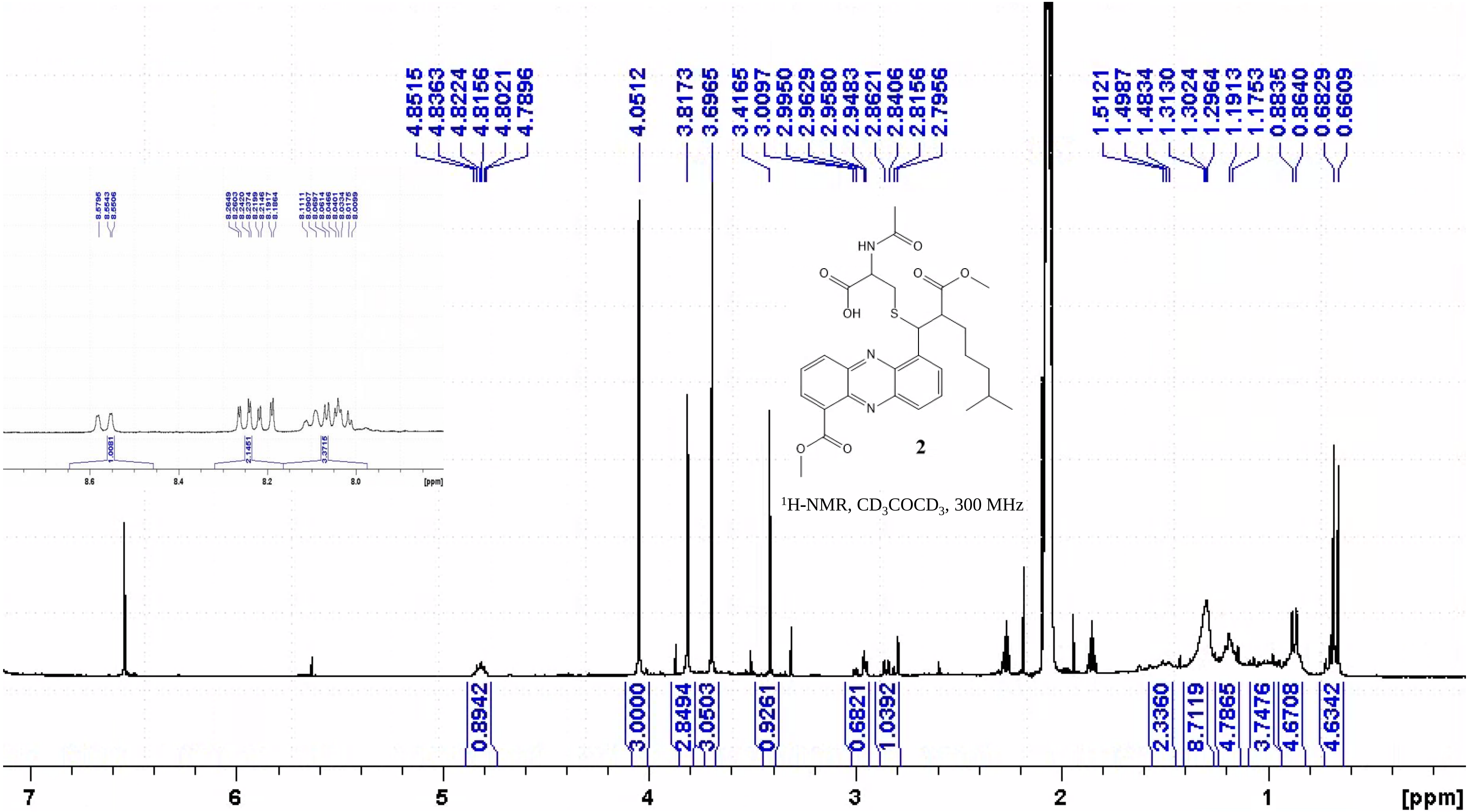

*Figure S19. ^{1}H-NMR of **2** in acetone-d_6 at 300K*

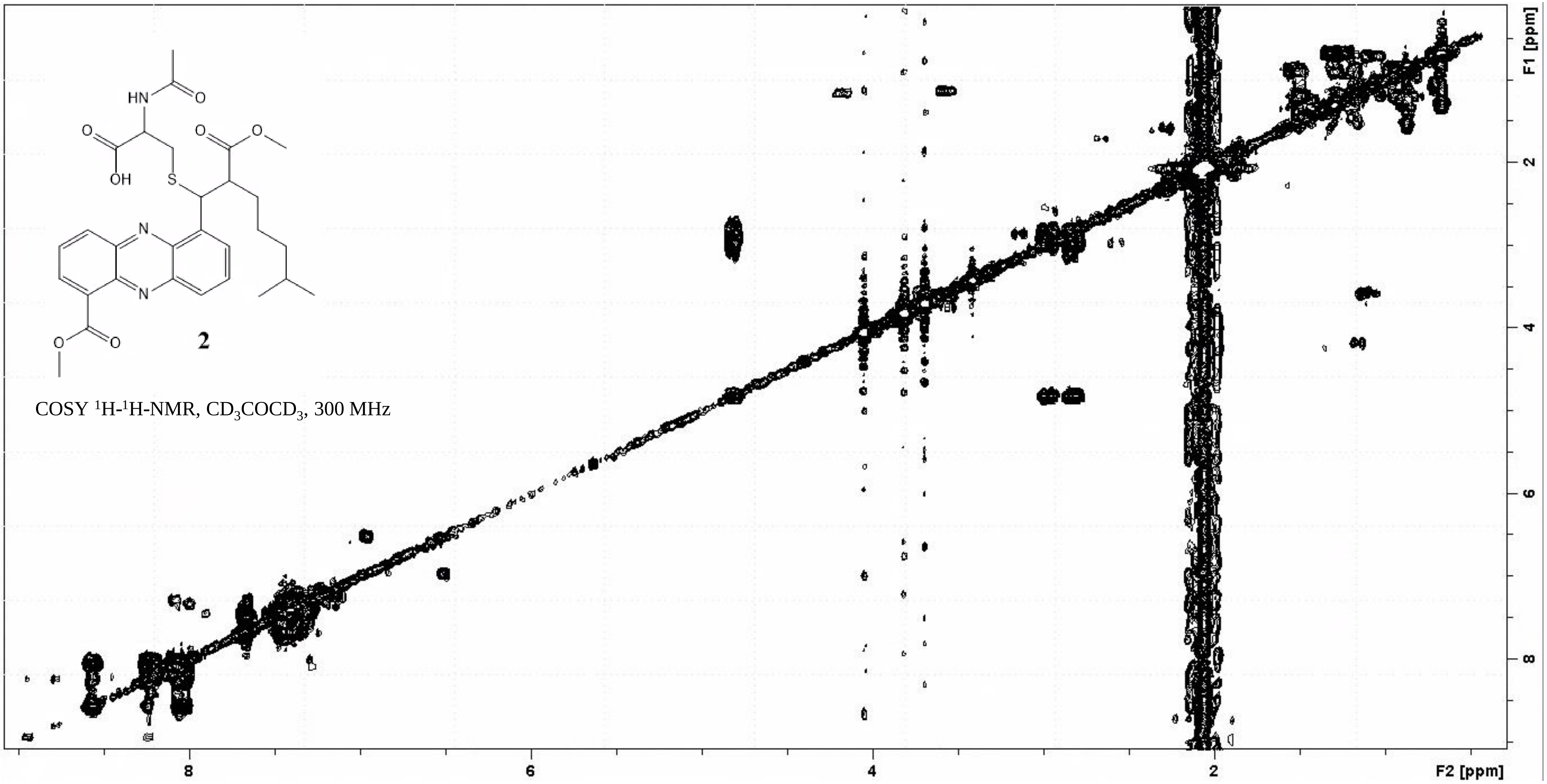

Figure S20. COSY of 2 in acetone-d_6 at 300K

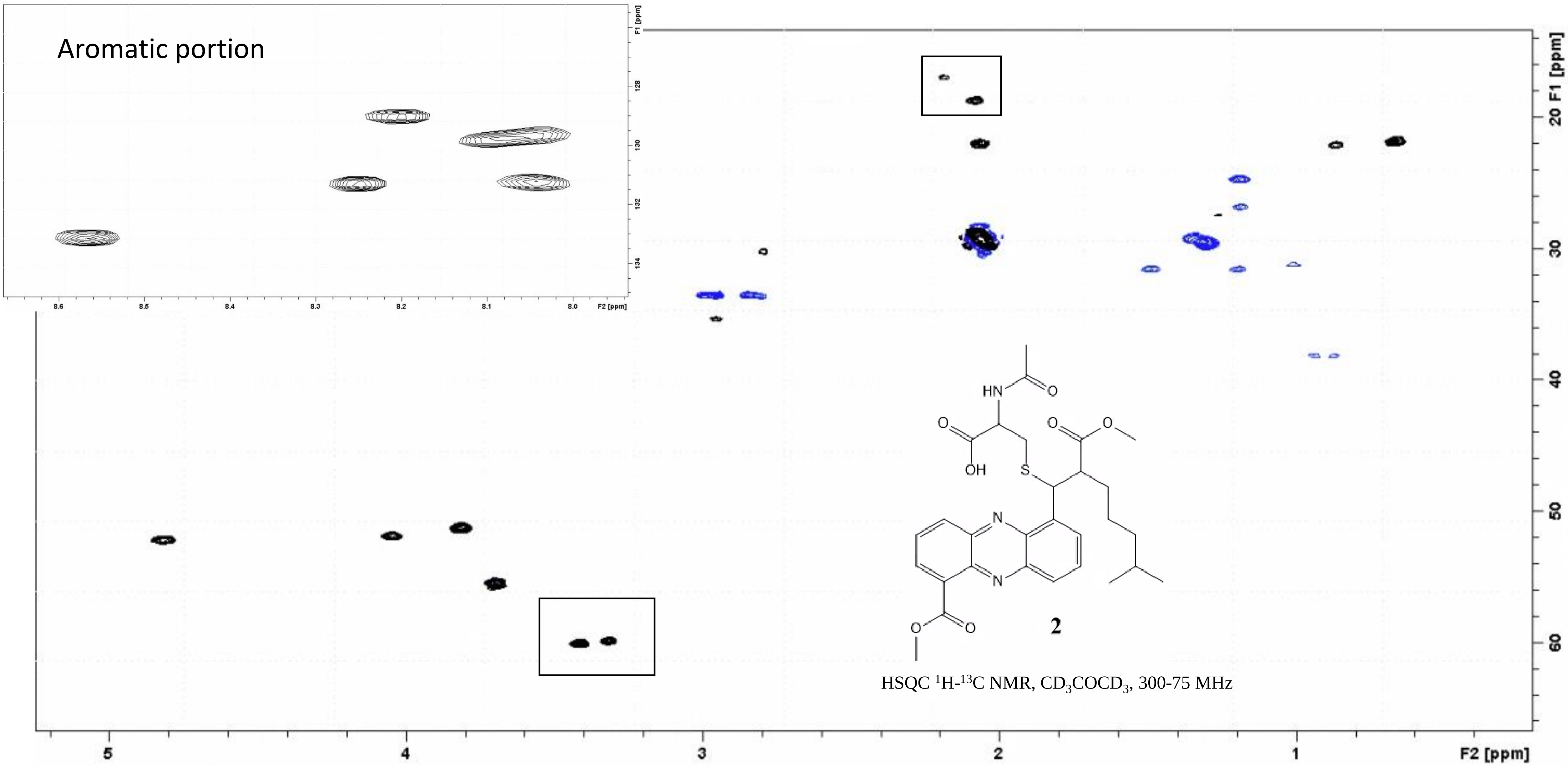

*Figure S21. HSQC of **2** in acetone-d_6 at 300K. Boxed: signals from unrelated impurities*

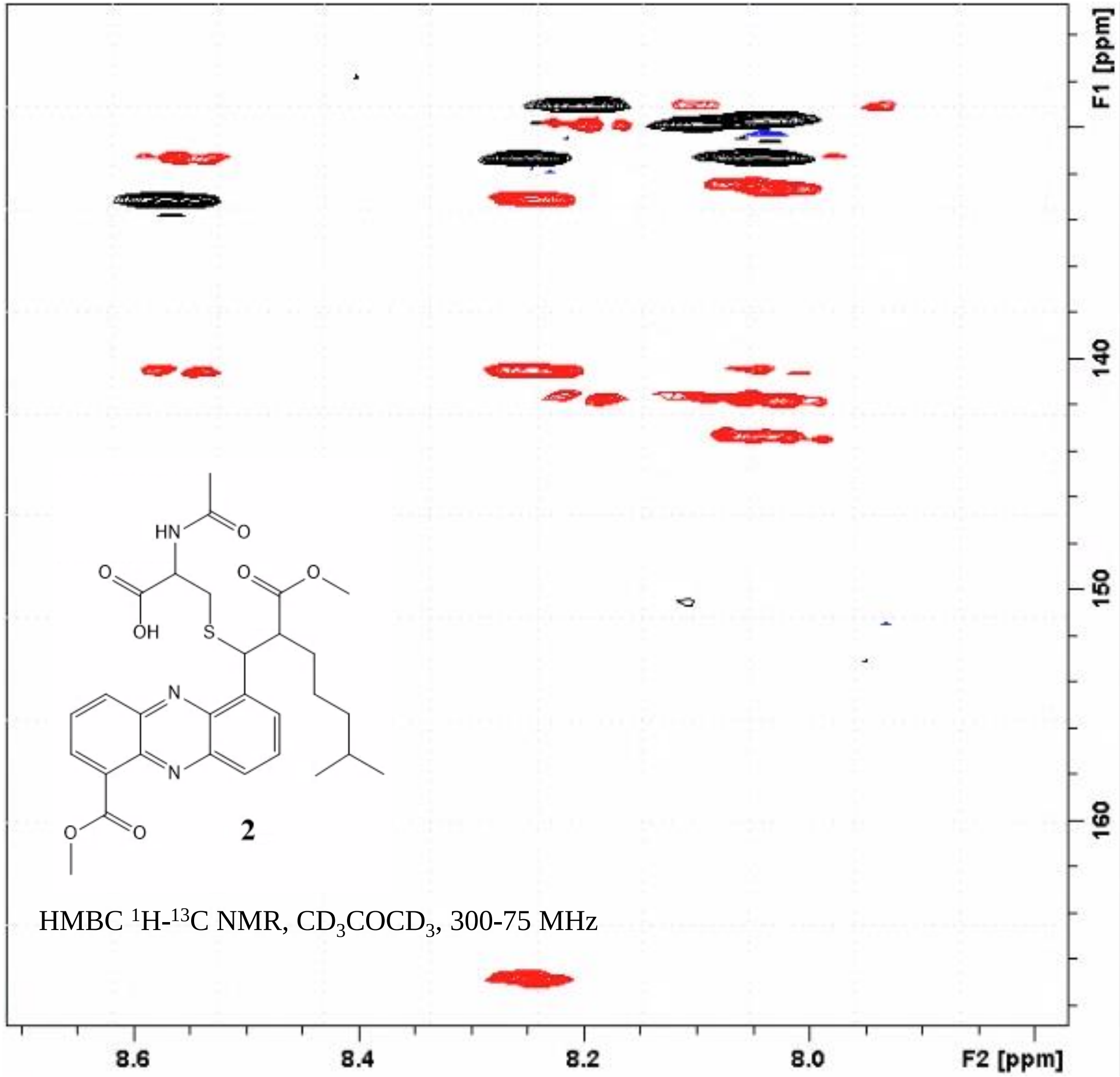

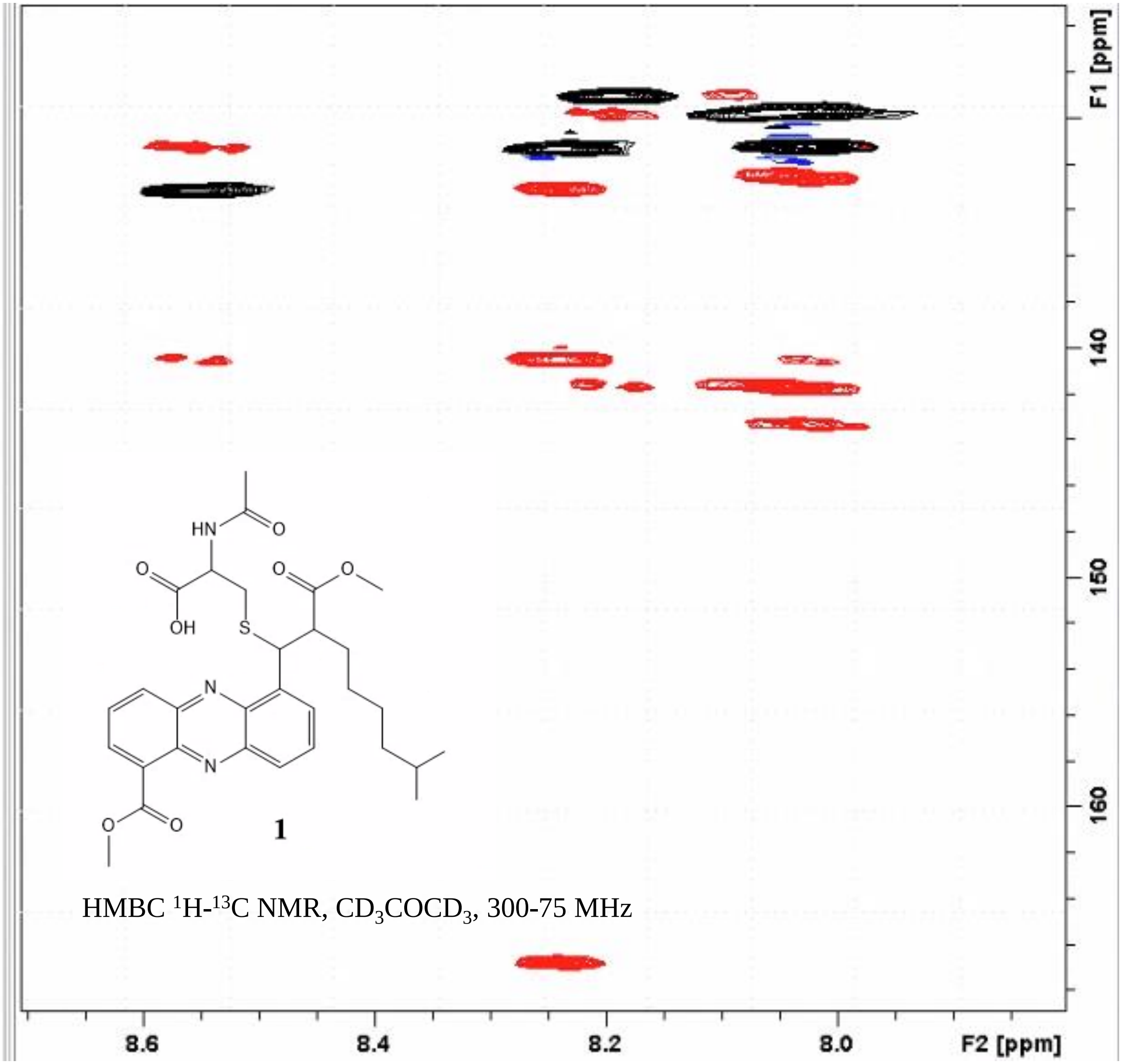

*Figure S22. HSQC (black) and HMBC (red) for **2** (left) and **1** (right) in acetone-d6 at 300K: aromatic portion*

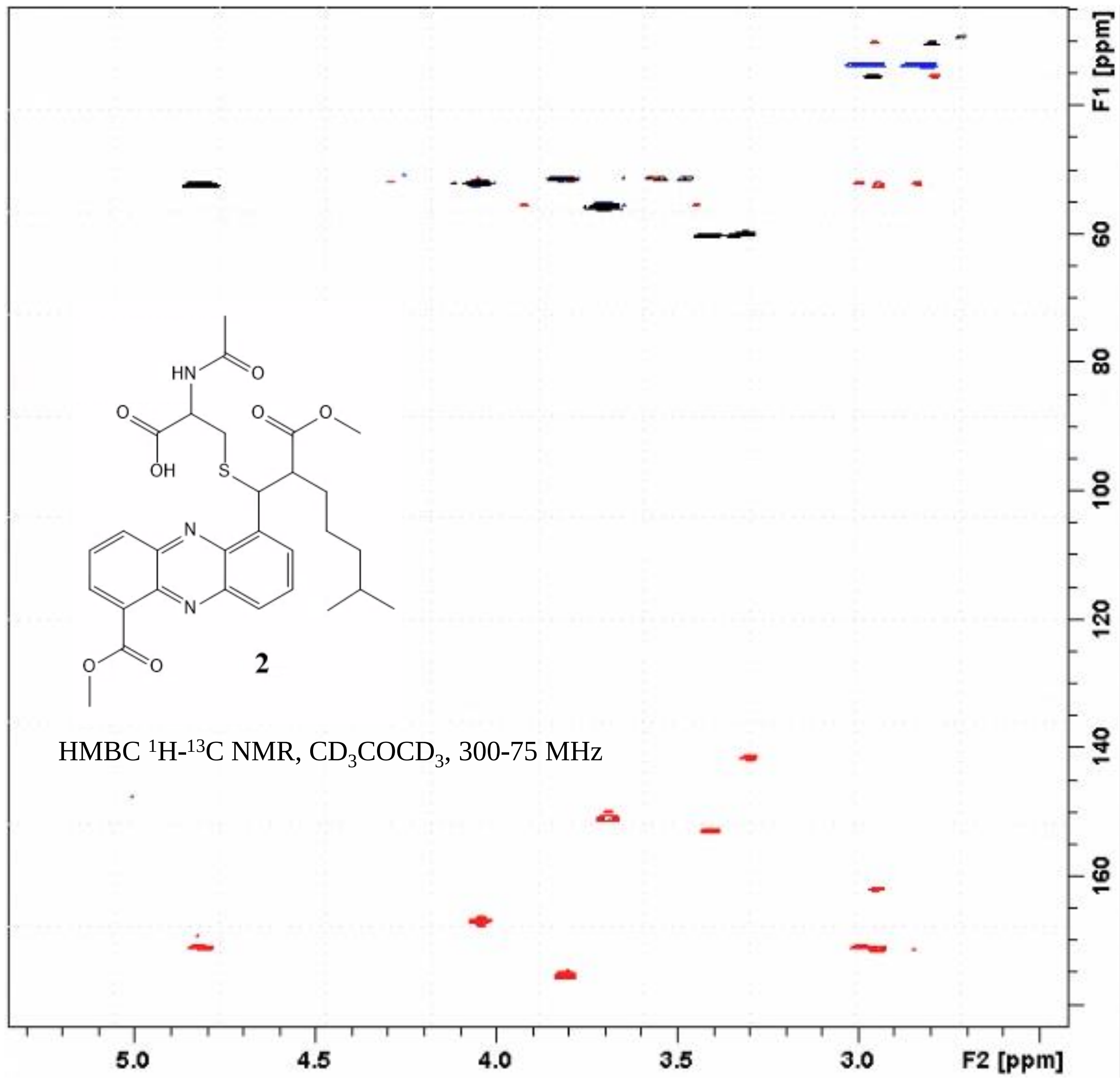

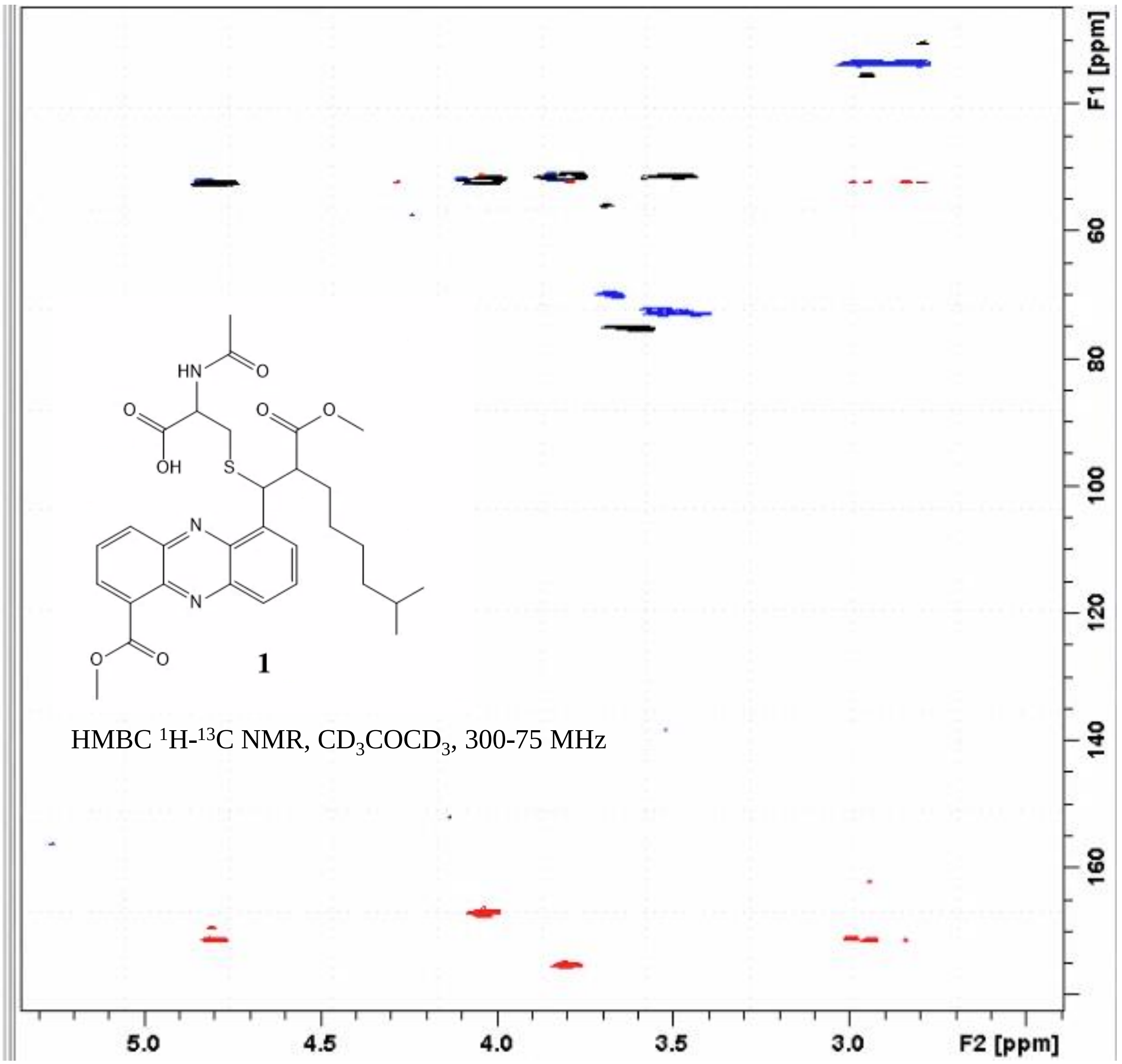

*Figure S23. HSQC (black) and HMBC (red) for **2** (left) and **1** (right) in acetone-d6 at 300K: central portion*

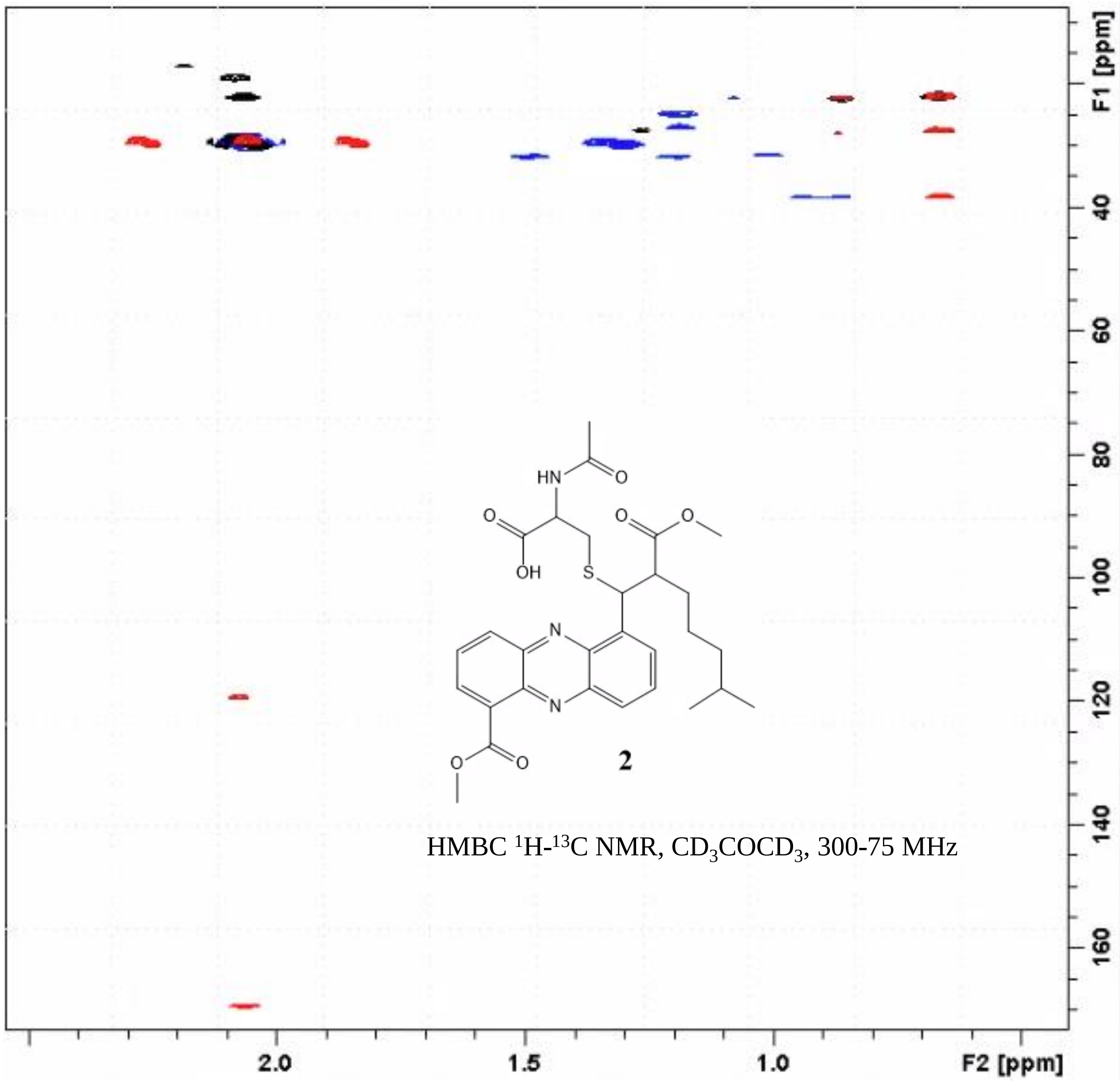

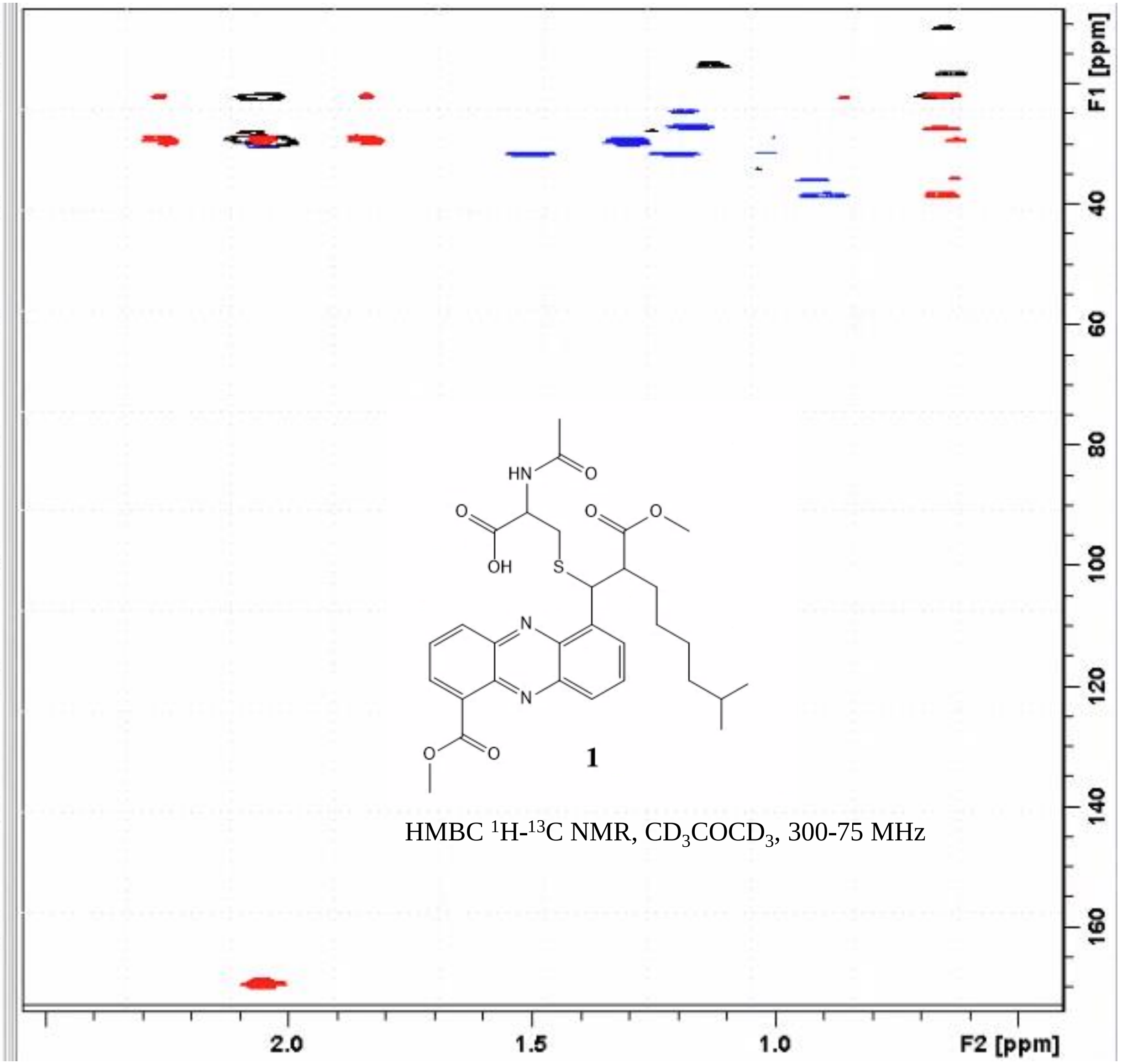

*Figure S24. HSQC (black) and HMBC (red) for **2** (left) and **1** (right) in acetone-d6 at 300K: aliphatic portion*

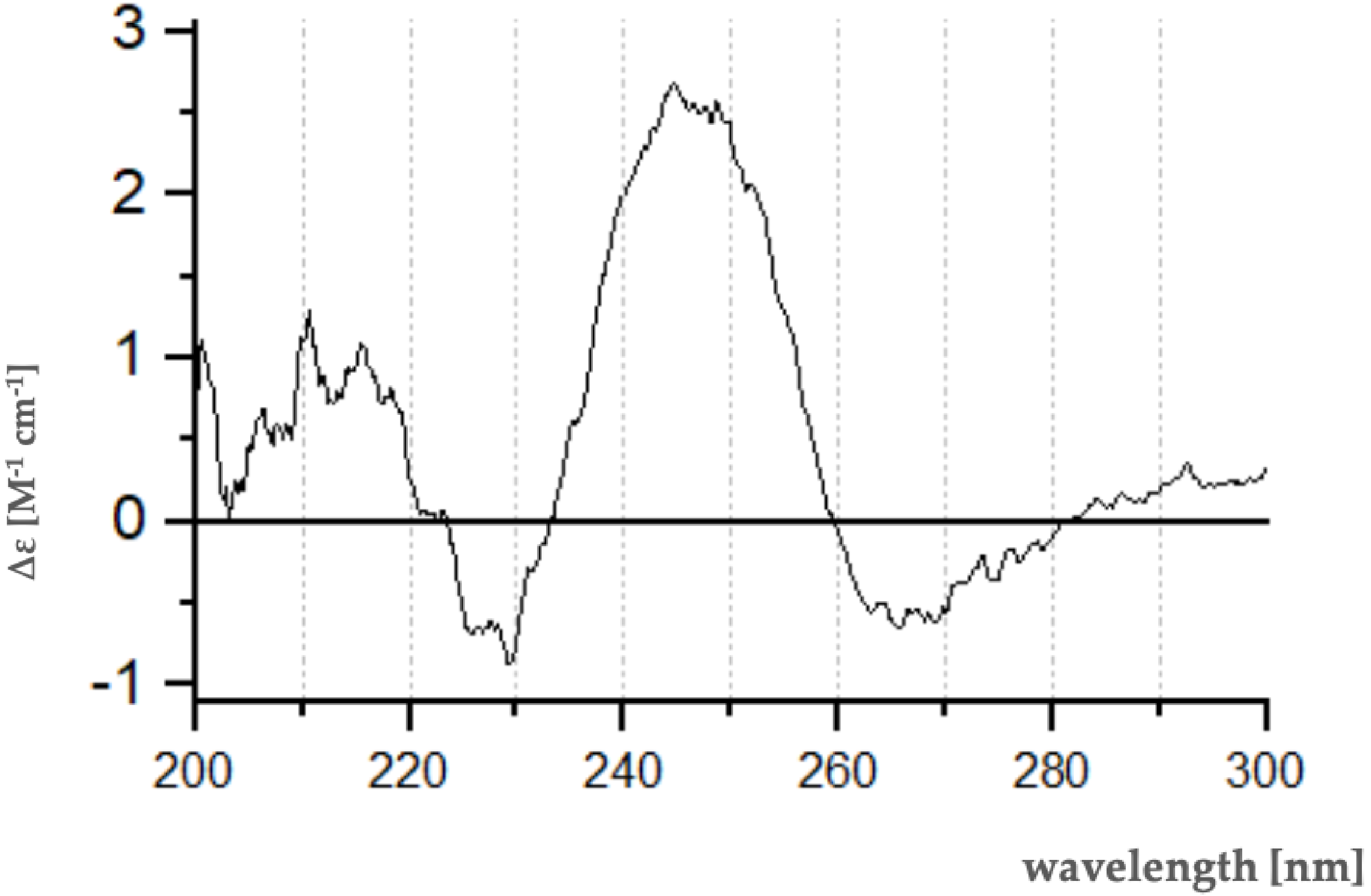

Figure S25. ECD spectrum of 1 in MeOH.

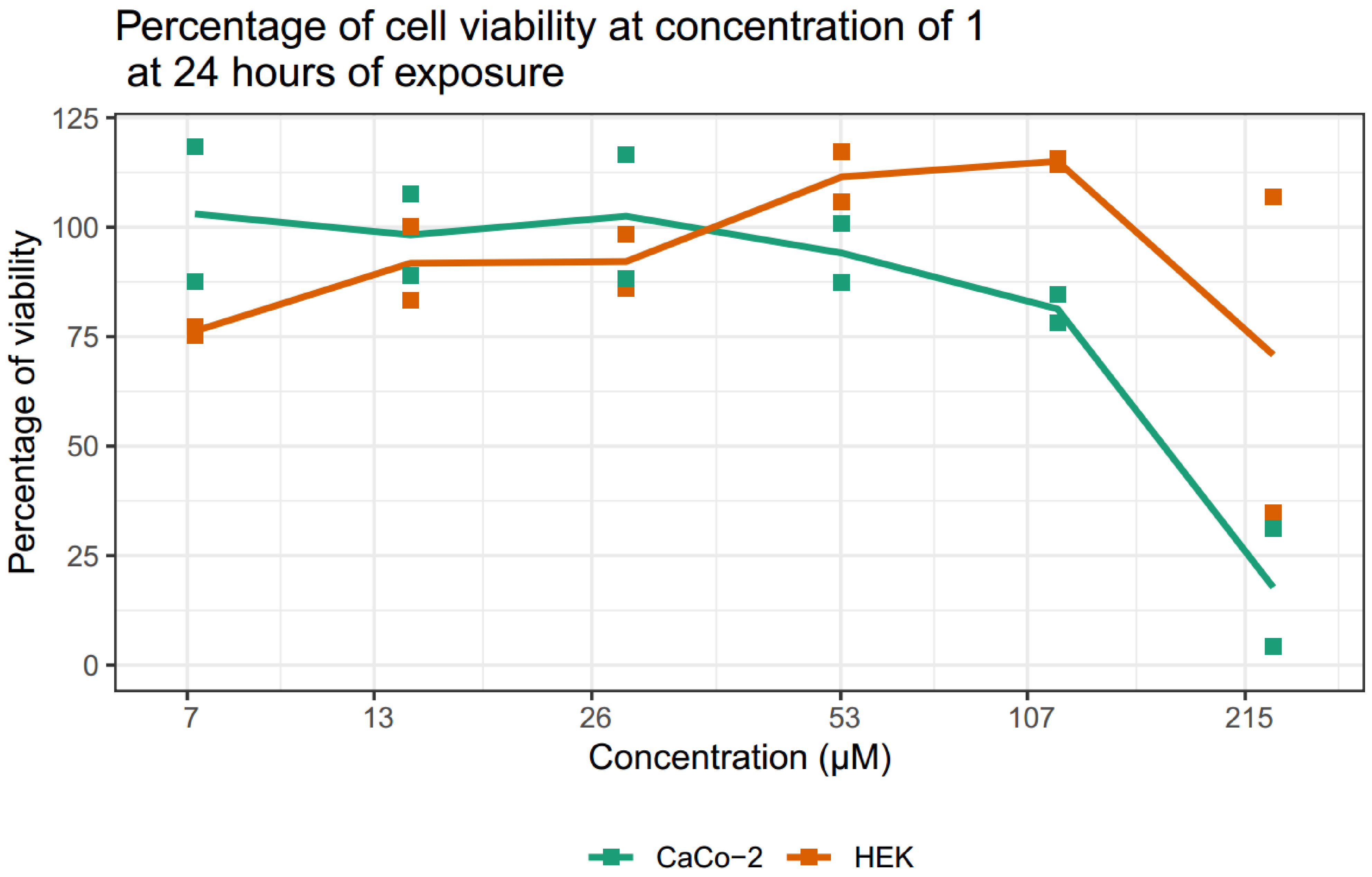

*Figure S26. Cytotoxicity of compound **1** on CaCo-2 and HEK cell lines. Percentage of cell viability at 24 h in the presence of **1**. Note log scale on x axis.*

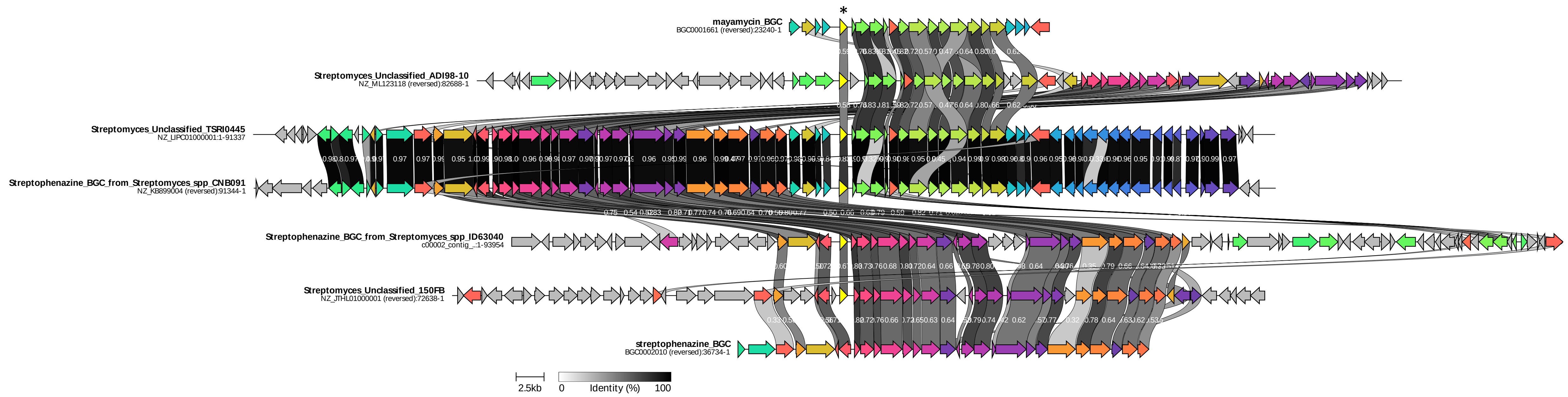

Figure S27. Comparison of streptophenazine BGC regions of strain ID63040 and of other Streptomyces streptophenazine BGCs from the antiSMASH database. The BGCs are anchored to the regulator ctg1_5 (yellow, marked with an asterisk) embedded in the ID63040 BGC. The mayamycin (top) BGC is also shown. Figure was generated with Clinker. White numbers on bands indicate identity of two genes.

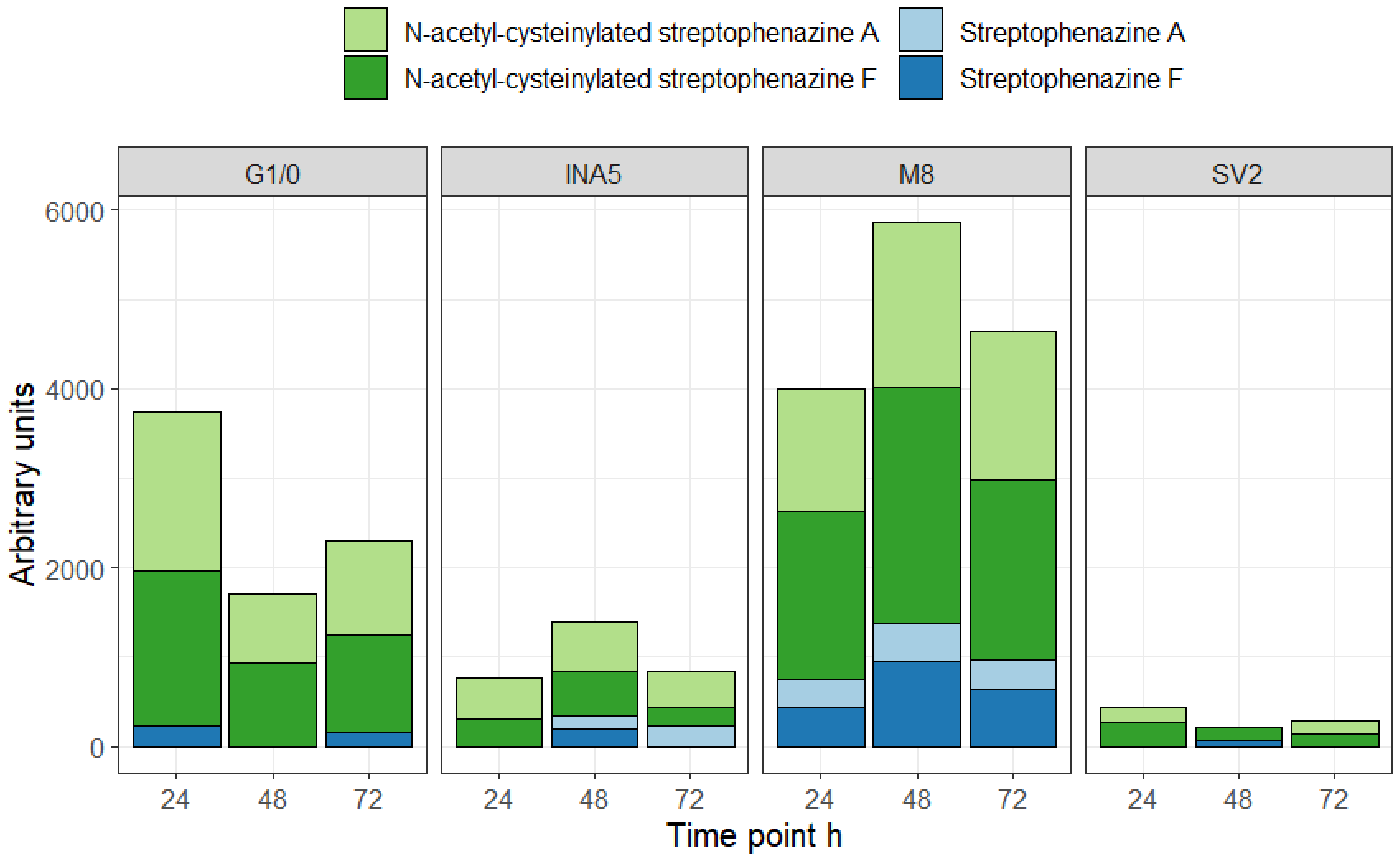

Figure S28. Abundances of 1'-(N-acetycysteinyl)-1'-deoxystreptophenazines A and F (greens) and streptophenazines A and F (blues) in four media (G1/0, INA5, M8 and SV2) at three timepoints (24, 48 and 72 h). Intensity of corresponding m/z signal on y-axis.

*Table S1. Calculated molecular formulae for parent mass, selected fragments and neutral losses of compounds **1** (top) and **2** (bottom). Ppm values >20 are highlighted in red. Experiments were conducted with high resolution instrument.*

m/z value	Neutral loss	C	H	N	O	S	Molecular formula	ppm	DBE
**584.2430**		30	38	3	7	1	C30H38N3O7S	4.9	13.5
	32.0235	1	4		1		CH4O	-84.77	0
552.2195		29	34	3	6	1	C29H34N3O6S	4.83	14.5
	131.0036	4	5	1	2	1	C4H5NO2S	-3.81	3
	163.0271	5	9	1	3	1	C5H9NO3S	-19.72	2
421.2159		25	29	2	4		C25H29N2O4	-6.85	12.5
	32.0268	1	4		1		CH4O	18.27	0
389.1891		24	25	2	3		C24H25N2O3	6.64	13.5
	27.9950	1			1		CO	3	2
361.1941		23	25	2	2		C23H25N2O2	6.91	12.5
	110.1111	8	14				C8H14	14	2
251.0830		15	11	2	2		C15H11N2O2	3.77	11.5

m/z value	Neutral loss	C	H	N	O	S	Molecular formula	ppm	DBE
**570.2310**		29	36	3	7	1	C29H36N3O7S	6.32	13.5
	32.0275	1	4		1		CH4O	40.13	0
538.2035		28	32	3	6	1	C28H32N3O6S	4.31	14.5
	131.0042	4	5	1	2	1	C4H5NO2S	0.77	3
	163.0317	5	9	1	3	1	C5H9NO3S	8.5	2
407.1993		24	27	2	4		C24H27N2O4	5.45	12.5
	32.0268	1	4		1		CH4O	18.27	0
375.1725		23	23	2	3		C23H23N2O2	4.35	13.5
	27.9947	1			1		CO	-7.67	2
347.1778		22	23	2	2		C22H23N2O2	5.32	12.5
	96.0952	7	12				C7H12	13.52	2
251.0826		15	11	2	2		C15H11N2O2	2.18	11.5

Table S2. Strains reported to produce streptophenazines and BGC sequence availability

Streptophenazines	Authors	Producer strain	Habitat	Sampling site	Host	BGC sequence availability
Streptophenazines A-H	Mitova, Lang et al. 2008	Streptomyces sp. strain HB202	marine	Germany, Baltic Sea	marine sponge Halichondria panicea	No
Revised structure of Streptophenazine A	Yang, Jin et al. 2011	Streptomyces sp. strain HB202	marine	Germany, Baltic Sea	marine sponge Halichondria panicea	No
Streptophenazines I-L	Bunbamrung, Dramae et al. 2014	Streptomyces sp. BCC21835	terrestrial	Thailand, Khao Khitchakut National Park, Chanthaburi province	soil	No
Streptophenazines I-K	Kunz, Labes et al.2014	Streptomyces strain HB202	marine	Germany, Baltic Sea	marine sponge Halichondria panicea	No
Streptophenazines M-O	Liang, Chen et al. 2017	Streptomyces sp. 182SMLY	marine	China, East China Sea	sediment	No
Streptophenazines P-R	Bauman, Li et al. 2019	Streptomyces sp. CNB-091	marine	USA, Florida Keys	jellyfish Cassiopeia xamachana	Yes

*Table S3. Hits for N-acetyl-cysteinylated streptophenazines **1** and **2** in public databases.*

m/z	MASST dataset ID	Source	Matched peaks	Cosine score
570.1783	MSV000083082:M1146-pKDB03.mzML	Streptomyces (NCBITaxon:1883)	14	0.78
584.1971	MSV000083082:M1146-pKDB03.mzML	Streptomyces (NCBITaxon:1883)	10	0.74
