# Supplementary material for: N-Acetyl-Cysteinylated Streptophenazines from Streptomyces
Source: J Nat Prod. 2022 Apr 15;85(5):1239–47. doi: 10.1021/acs.jnatprod.1c01123 (PMC9150181; doi:10.1021/acs.jnatprod.1c01123)

**Instrumentation:** ^1^H and ^13^C NMR spectra were recorded with a Bruker 300 MHz Avance II spectrometer

**Acquisition software:** Bruker Topspin 3.2

**Processing program:** Bruker Topspin 3.2

**Operating Frequencies:** 300 MHz ^1^H, 75 MHz ^13^C


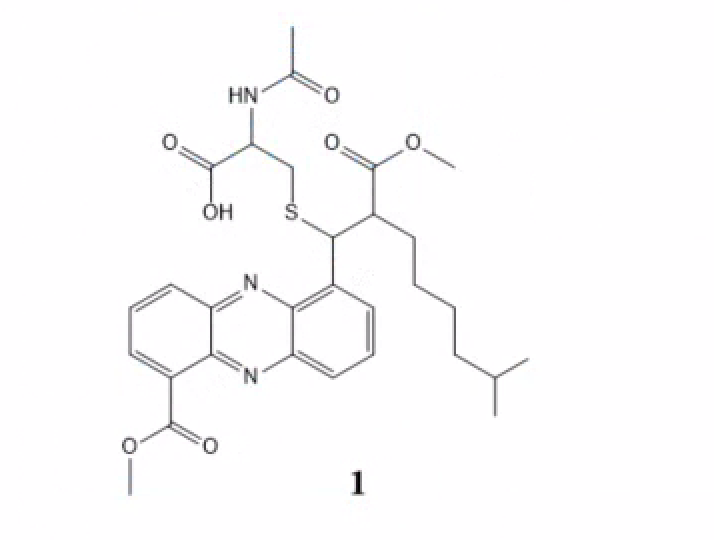


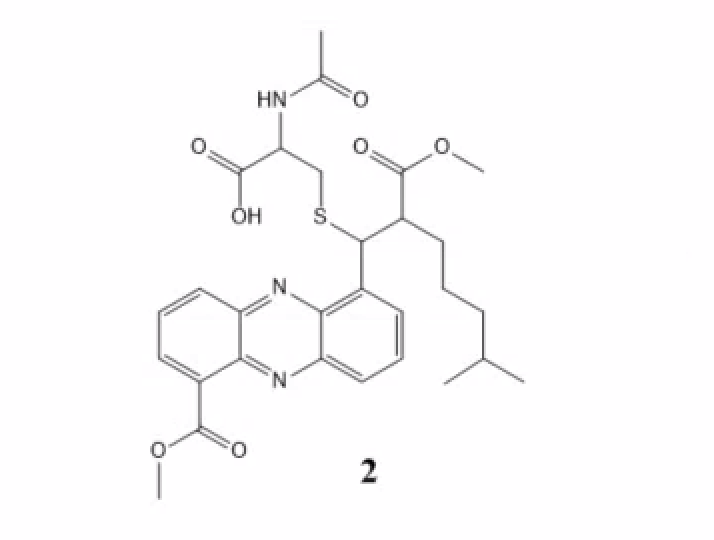

Supplement: Supplementary file 2 — np1c01123_si_002.zip [file np1c01123_si_002.zip › FID for publication/TEXT.docx]
